# Supplementary material for: Landscape of Bone Marrow Metastasis in Human Neuroblastoma Unraveled by Transcriptomics and Deep Multiplex Imaging
Source: Cancers (Basel). 2021 Aug 26;13(17):4311. doi: 10.3390/cancers13174311 (PMC8431445; doi:10.3390/cancers13174311)
Supplement: Supplementary file 1 [file cancers-13-04311-s001.zip › cancers-1331004 - SUPPLMENTARY/cancers-1331004-supplementary-final_STM.pdf]

cancers

Supplementary Materials: Landscape of Bone Marrow Metastasis in Human Neuroblastoma Unraveled by Transcriptomics and Deep Multiplex Imaging

Daria Lazic, Florian Kromp, Fikret Rifatbegovic, Peter Repiscak, Michael Kirr, Filip Mivalt, Florian Halbritter, Marie Bernkopf, Andrea Bileck, Marek Ussowicz, Inge M Ambros, Peter F Ambros, Christopher Gerner, Ruth Ladenstein, Christian Ostalecki and Sabine Taschner-Mandl

1. Methods

1.1. Biomarker Identification by Data Mining

DTC-associated biomarkers were identified based on data mining of previously generated RNA-seq datasets and proteomics data, and guided by public databases. The following prioritization scheme was employed:

1.1.1. Differential Gene Expression Analysis

RNA-seq data (GEO repository available under accession number GSE94035) of primary tumors (n = 16), enriched bone marrow-derived diagnostic (n = 22) and relapse DTCs (n = 20), and the corresponding bone marrow-derived MNCs (n = 28) of in total 53 stage M neuroblastoma patients was processed as previously described [1] and used for the identification of potential DTC biomarkers. Genes with significantly higher (DEseq2 [2], FDR-adjusted $p \leq 0.001$, log2FC ≥ 4) transcript levels (FPM, fragments per million) in DTCs as compared to bone marrow-derived MNCs were selected (n = 1,594) and further filtered for those with an equal or higher transcription (DEseq2, FDR-adjusted p: 0.01 ÷ 0.7, log2FC ≥ 0) at the time point of relapse as compared to diagnosis (n = 921).

All 53 patients were annotated for neuroblastoma genetic aberrations (Table S4) and their correlation with the mRNA transcript levels (FPM, fragments per million) of DTC markers (CD56, GD2, CD276 and FAIM2) was evaluated using the Wilcoxon-Mann-Whitney test with FDR-correction (Figure S15).

1.1.2. Protein Databases and Literature Search

The remaining genes were manually annotated with the cellular location of the encoded protein according to protein databases UniProt [3] and The Human Protein Atlas [4], and only proteins localized on the cell membrane by at least one database were further considered (n = 134, Table S5).

Detailed literature search using the search terms [neuroblastoma], [tumor] and [metastasis] was carried in the PubMed database (pubmed.ncbi.nlm.nih.gov) resulting in 99 candidates (Table S6), from which five (TAG1, DCLK1, FAIM2, PRAME and TACC2) were selected based on detailed examination of available literature and commercial availability of respective antibodies.

1.1.3. Proteomics Data

Proteomics data of eight peripheral-nerve-associated fibroblasts, 3 in-house established patient-derived neuroblastoma cell lines (STA-NB-10, STA-NB-2, STA-NB-7) and 6 corresponding neuroblastoma primary tumors was previously generated [5] and is available on the ProteomeXchange Consortium (proteomecentral.proteomexchange.org) with the dataset identifier PXD018267. The proteomics dataset was used (Table S7) to confirm the expression of the five candidates selected above as well as seven other

biomarkers (CD56, NCAM-L1, PD-L1, VIM, PROM1, B7-H3 and PD1), which were added based on their relevance in neuroblastoma as previously reported [6–9].

1.2. Biomarker Validation

1.2.1. Cytospin Slide Preparation

50,000 cells (cell lines) or 250,000 cells (spike-in and patients samples) were applied onto poly-L-lysine hydrobromide (PLL) (Sigma Aldrich) -coated microscope cover glasses (24 × 60 mm, Assistent) using filter paper (4 mL, CytoSepTM) and funnel chamber (4 mL, CytoSepTM) of a Hettich cyto-centrifuge (Hettich). Three different centrifugation and fixation methods were tested in the present study (Table S12, Figures S3b and S4a). The optimized protocol (PFA-AC) for processing patient samples is detailed in Table S12 and involves paraformaldehyde (PFA) followed by acetone (AC) fixation.Chemicals used for fixation, acetone and 4% PFA, were ordered from Carl Roth GmbH. Slides were dried for 2 min after fixation and stored at − 80°C until further analyses.

1.2.2. IF Staining

Antibodies (Table S14) were diluted in 2% BSA/PBS. Slides were incubated with primary antibody solutions for 1 h at room temperature, washed in PBS twice followed by secondary antibodies for 1 h at room temperature. After washing, slides were incubated with the nuclear stain DAPI (2 µg/mL) for 2 min and covered with antifade medium Vectashield (Vector Laboratories).

1.2.3. Validation Procedure

DTC-related biomarkers were validated based on an intuitive validation procedure (Figure S3a). First, IF-staining of individual biomarkers was performed on neuroblastoma cell lines prepared with the AC and PFA based protocol (Table S12, Figure S3b). Thereafter, slides were assessed visually using a Zeiss Axioplan two microscope in five criteria (nuclear morphology, background noise, cell debris, staining intensity, staining quality) to evaluate the impact of the respective sample preparation protocol (AC or PFA) on cell morphology and staining quality. Scores from one to five were assigned to each criterion with five corresponding to the best result. Accordingly, the maximum score for one slide was 25. Scores for all five neuroblastoma cell lines, incubated with the corresponding antibody, were summed up for each fixation method separately, and a mean score was calculated as a qualitative metric. Although AC outperformed the PFA based protocol in terms of staining intensity, staining quality and background noise, PFA is a gentler fixation reagent, which better maintains nuclear morphology and leads to less cell debris. This is shown by separate scores for staining quality and morphology (Table S13). Antibodies with a mean overall score below 13 for both AC and PFA based fixation were considered invalid and not further validated. For all other antibodies, images of the slides prepared with the better sample preparation protocol (higher mean score) were acquired using the automated scanning system, Metafer 4 (software version V3.11.8 WK, Metasystems) and 63 × magnification (Figure S3c).

Antibodies that were successfully validated on neuroblastoma cell lines, were additionally tested on two cytospin samples of neuroblastoma cell lines and bone marrow-derived MNCs or peripheral blood-derived MNCs (for validation of PD-L1, PD-1). Contrary to neuroblastoma cell lines, bone marrow- and peripheral blood-derived MNCs did not tolerate the AC protocol, which led to the establishment of the PFA-AC protocol to combine the benefits of AC and PFA based sample preparation (Table S12, Figure S4a). Slides were then visually inspected and imaged automatically as above (Figure S4b).

For sequential IF-staining by MELC, antibodies, which passed the validation procedure, were combined with already validated antibodies specific to bone marrow hematopoietic and mesenchymal cells (Table S14). Staining sequence and panel were refined in several pilot MELC rounds and finally resulted in a 20-plex biomarker panel (Table 1, Figure 1e,f).

1.3. Interphase Fluorescence in Situ Hybridization (iFISH)

The MELC pre-processed sample BM 1.1 was fixed in 4% paraformaldehyde at 4°C overnight for subsequent analysis by iFISH. iFISH was performed as previously described [10]. Predigestion of cells was carried out in 0.005% pepsin in 0.01 NHCL for 25 min. Since the sample originated from a patient with a chromosome 17q gain, a labeled 17q-specific probe (XL Iso (17q), Metasystems probes) was used. Denaturation was performed at 80°C. Nuclei were counterstained with nuclear stain DAPI (2 µg/mL) for 2 min and covered with antifade medium Vectashield (Vector Laboratories). Slides were imaged with the Zeiss Axioplan 2 microscope and the ISIS software (v5.7.4, Metasystems).

1.4. DeepFLEX

1.4.1. Normalization

Mutually exclusive marker pairs (Table S15) were selected based on biological knowledge and a data-driven approach using singular value decomposition, as described [11].Inference is performed by three different clustering methods: Sparse Subspace Clustering, Gaussian Mixture Model, k-Means. Local background levels were then inferred for each field of view and each intensity feature, separately. For the purpose of visualization, global background values were inferred based on all fields of view of all samples combined. Guided by generated scatter plots, we selected the background level predicted by sparse subspace clustering ($\sigma = 0$) as the most appropriate threshold. If no positive signals were present in the analyzed field of view for a certain marker by visual inspection, the respective background level was set to the maximum intensity value.
Subsequently, all values below the background level were randomly set within a range between 0 and 0.02, while all values exceeding the background level (corresponding to signals) were linearly scaled to a range between 0.02 and 1. Thereby, influence of background variation on the subsequently applied single-cell analysis was eliminated, while foreground signals were stretched to a larger dynamic range.

Morphological features were linearly scaled between 0 and 1. Upon RESTORE normalization and scaling, batch effects were successfully removed (Figure S2d, bottom).

2. Figures

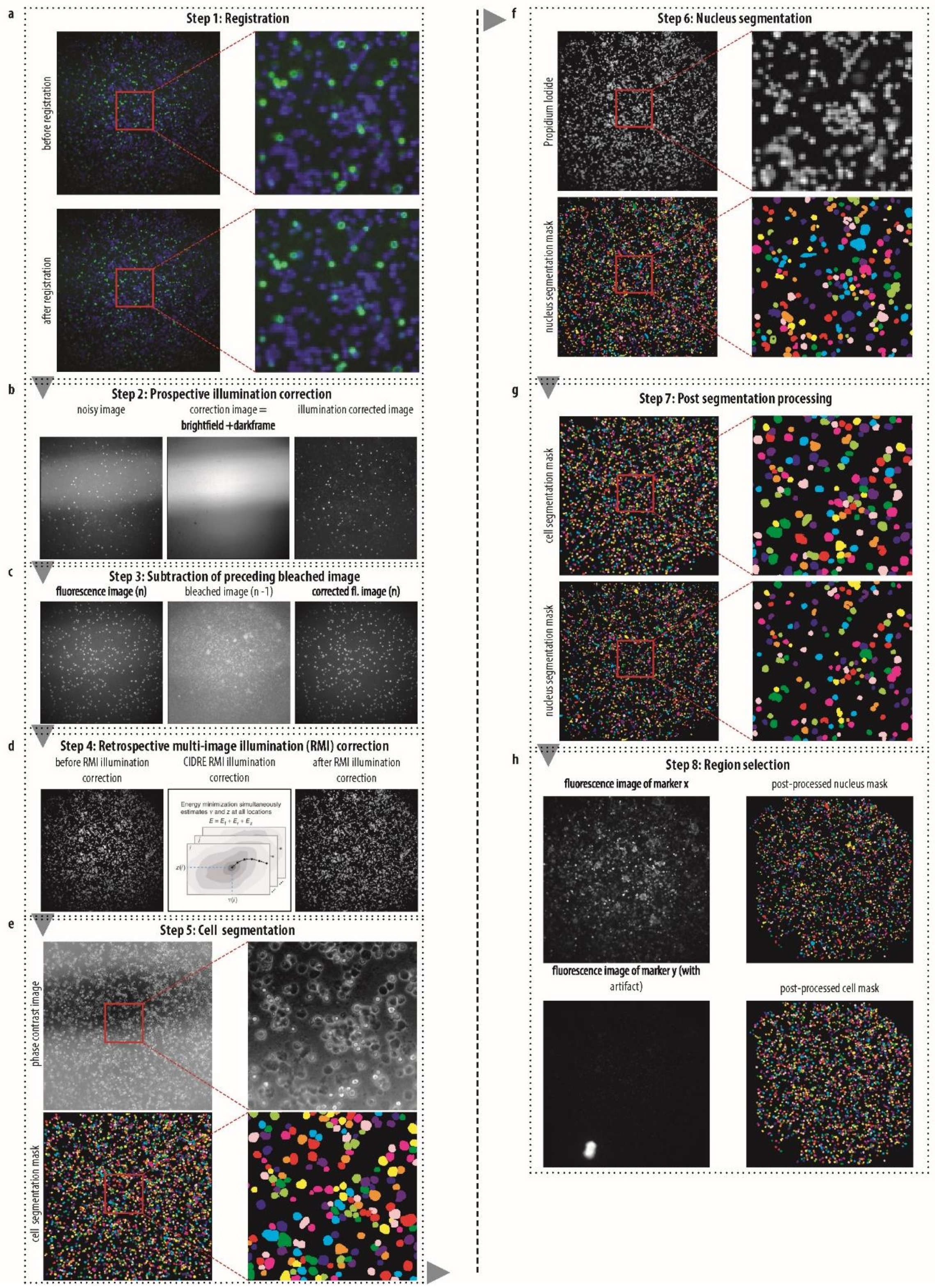

Figure S1. Deep Flex (Image Processing and Segmentation). (**a**) Image registration by fourier transformation followed by multiplication of fourier transforms and product inversion (IDFT) to obtain cross-correlation. Peak is first located at pixel-level and subsequently on sub-pixel level to compute the translative offset between images. (**b**) Prospective illumination correction by subtraction of brightfield and dark field image from fluorescence and bleaching image of each antibody. Prospective illumination correction eliminates bright stripe in the centre, but not "vignetting" (reduced image brightness towards image periphery). (**c**) Accumulative background noise caused by residual post-bleaching signals eliminated by subtracting post-bleaching image of preceding biomarker from fluorescence image of current biomarker. (**d**) Retrospective

multi-image illumination correction by the open-source tool "CIDRE" [12],which is based on a regularized energy minimization and builds the correction function by using all acquired images. (**e**) Cell segmentation is performed on phase contrast images of every marker by a Mask R-CNN architecture. (**f**) Nucleus segmentation is performed on the fluorescence image of marker propidium Iodide by a Mask R-CNN architecture [13]. (**g**) Only cells which are present in both nucleus and cell segmentation mask are kept. (**h**) Regions of homogeneous illumination are selected and artifacts are excluded for each channel by a user.

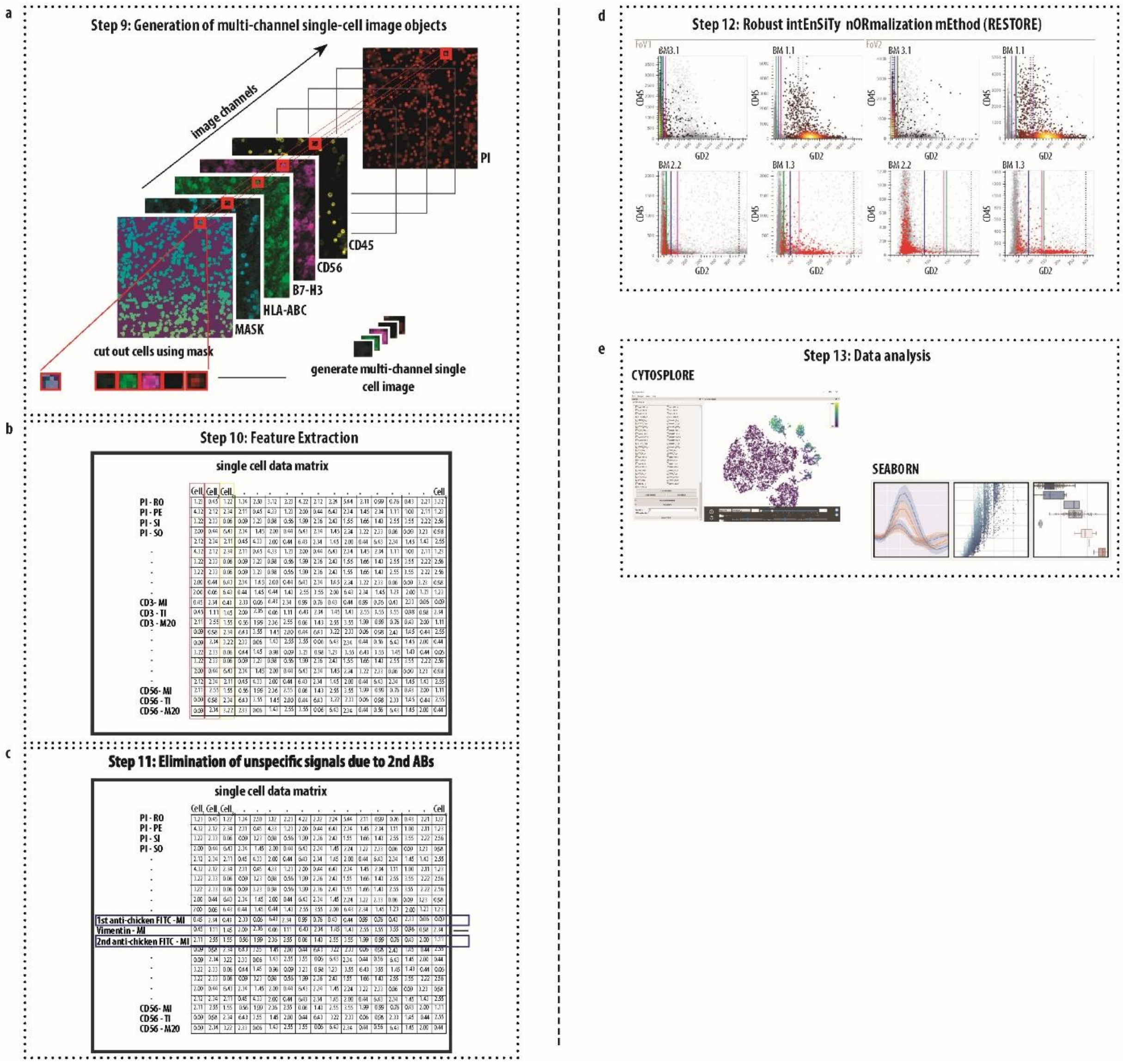

Figure S2. DeepFLEX (Feature-Extraction, Normalization and Single-Cell Analysis). (**a**) Multi-channel single-cell image objects are generated based on a nucleus and cell segmentation mask. Three masks are stored: nucleus mask, cell mask, cytoplasm/membrane mask (cytoplasm mask - nucleus mask). (**b**) Morphological and intensity features for cell, nucleus and cytoplasm/membrane of each cell are extracted. (**c**) Division of 2nd secondary antibody by 1st secondary antibody to reduce background- and amplify specific signals, thereby increasing SNR (signal to noise ratio). SI, size; SO, solidity; PE, perimeter; RO, roundness; MI, mean intensity; TI, total intensity; M20, mean of the highest 20% of pixel values. (**d**) Top: Inference of local background intensity levels (dotted lines) for each feature and each field of view (FoV) separately using mutually exclusive marker pairs, based on the method RESTORE [11]. For the purpose of visualization, global background values (solid line) are inferred based on all FoVs of all samples. Inference is performed by three different clustering methods: Sparse Subspace Clustering (green), Gaussian Mixture Model (blue), k-Means (magenta). Signal intensities are then divided by the background intensity level inferred by Sparse Subspace Clustering. For markers, for which no positive cells exist in one FoV, signal intensities are divided by maximum value (black, dotted line). Bottom: Batch effect before and after RESTORE. (**e**) Integration of seaborn [14] and Cytosplore [15] for single-cell analysis.

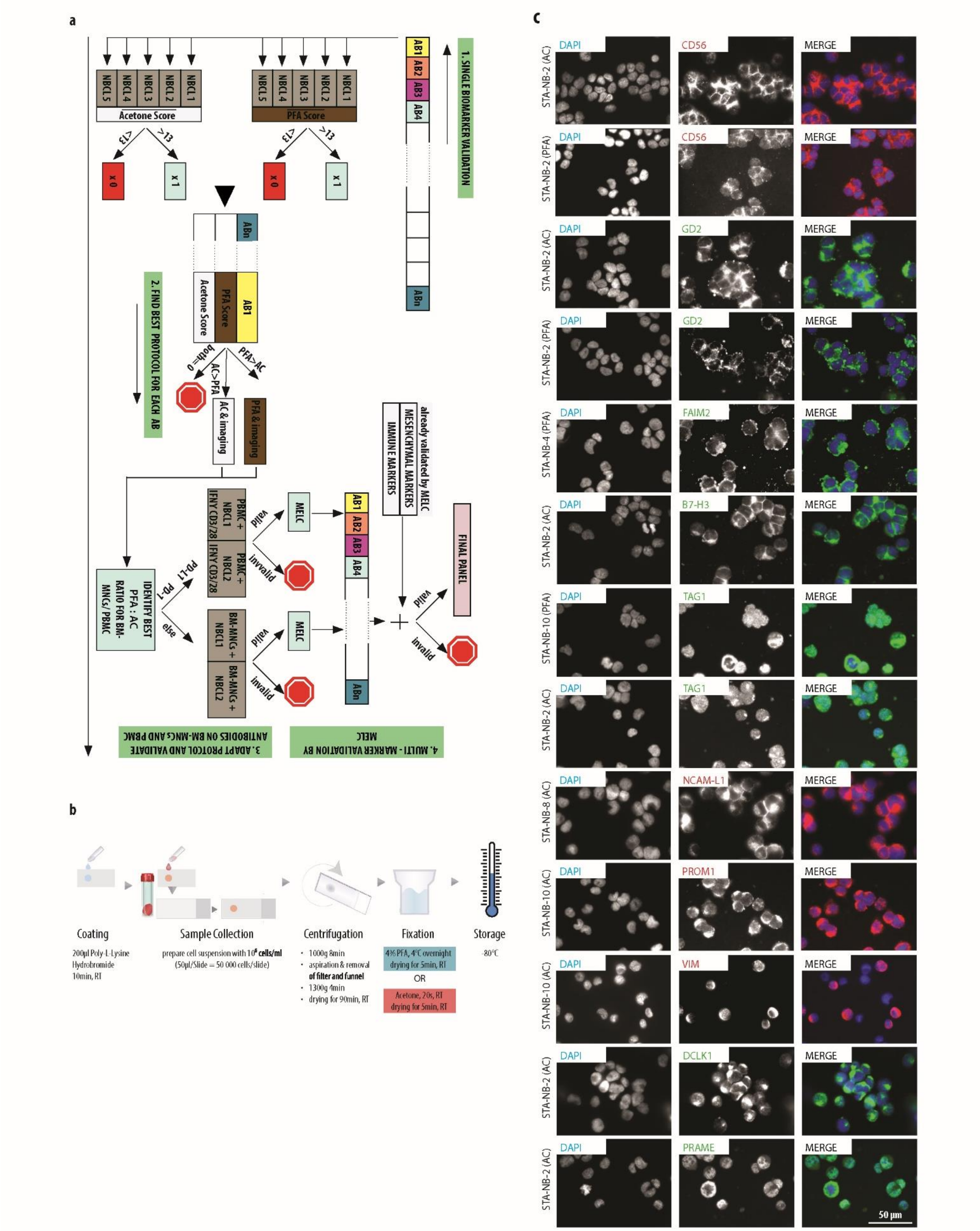

Figure S3. Validation of antibodies against DTC-related biomarkers on neuroblastoma cell lines (NBCL). (**a**) Overview of biomarker validation. 1) Single biomarker validation on 5 NBCL samples prepared with an AC (acetone)- and a PFA (paraformaldehyde)-based fixation protocol. 2) Identification of a better fixation method for each antibody and exclusion of non-valid antibodies. 3) Combination of PFA and AC and adaptation of protocol according to evaluation results for subsequent preparation of samples of NBCL spiked into bone marrow-derived MNCs (BM-MNCs) or peripheral blood-

derived MNCs (PBMC). Single biomarker validation on these samples prepared with adapted protocol. 4) Multi-marker validation of functional biomarkers by MELC and identification of a final biomarker panel. (**b**) Two protocols were compared. Protocol PFA (blue rectangle) employs PFA-based fixation. Protocol AC (red rectangle) employs acetone-based fixation. Coating is required due to the application of microscope cover glasses, which present a different surface treatment as compared to conventional microscope slides. (**c**) Representative IF images showing comparison of AC- versus PFA-fixed NBCL STA-NB-2 stained with CD56 (red) and GD2 (green); potential DTC biomarkers TAG1 (green), DCLK1 (green), FAIM2 (green) and PRAME (green) on one representative NBCL sample prepared with the better protocol (for TAG1 both PFA- and AC-fixed samples are shown, since PFA score was slightly higher than AC score); immune checkpoint molecule B7-H3 (green), therapeutic target NCAM-L1 (red), and mesenchymal markers VIM(red) and PROM1 (red) on one representative NBCL sample prepared with the better protocol. Nuclei were counterstained with DAPI (blue). Stainings are ordered by their quality with the best on top.

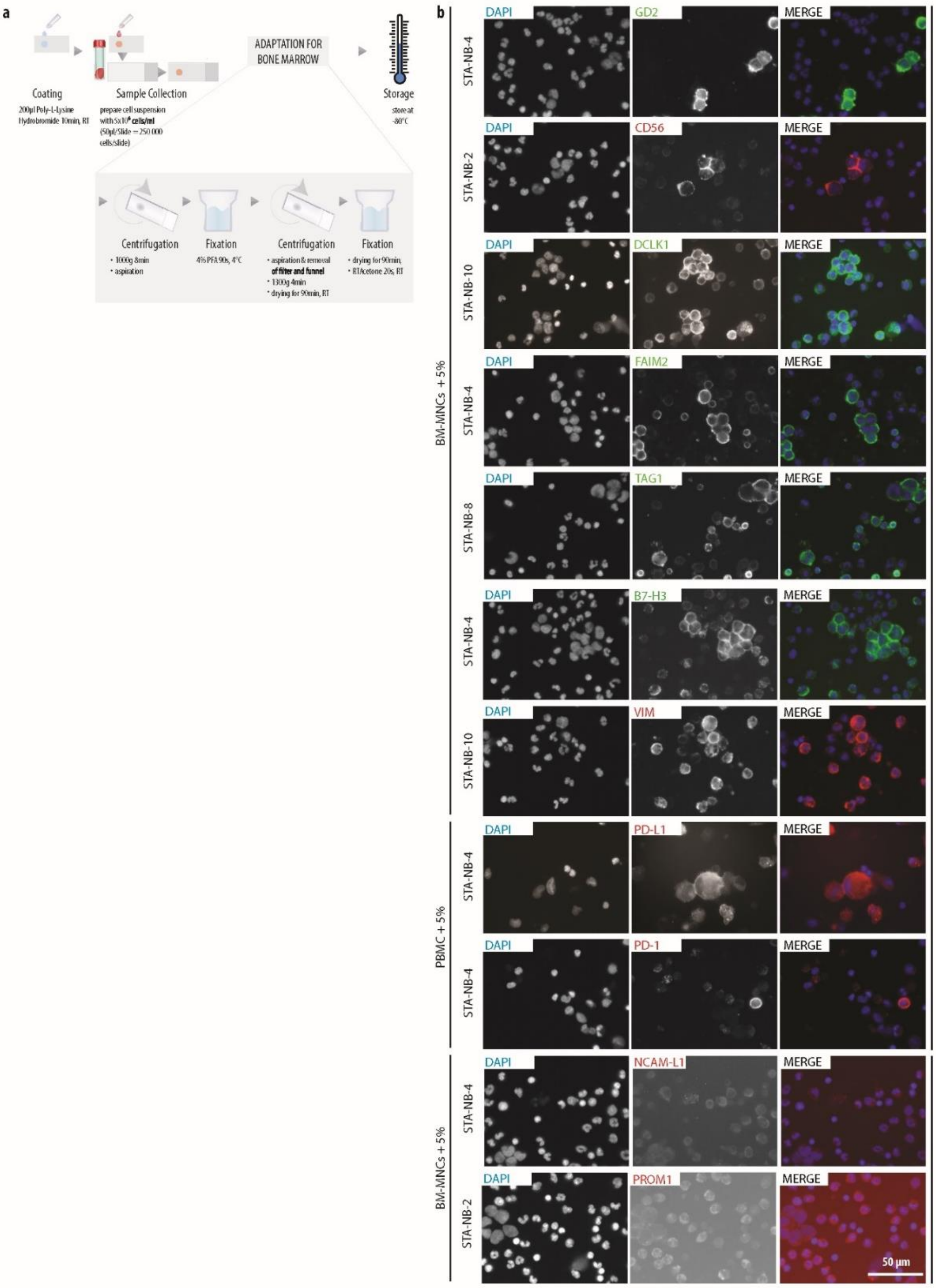

Figure S4. Validation of antibodies against DTC-related biomarkers on neuroblastoma cell lines (NBCL) spiked into bone marrow derived mononuclear cells (BM-MNCs) or peripheral blood-derived mononuclear cells (PBMC). (**a**) Schematic

representation of the PFA-AC protocol with adapted fixation and centrifugation method to combine benefits of AC and PFA based sample preparation, while preserving the integrity of BM-MNCs and PBMC. (**b**) Representative IF staining images of biomarkers GD2 (green) and CD56 (red), potential novel biomarkers TAG1 (green), DCLK1 (green) and FAIM2 (green), immune checkpoint molecule B7-H3 (green), therapeutic target L1-CAM (red), and mesenchymal markers VIM (red) and PROM1 (red) on samples of NBCL mixed with BM-MNCs (1:20). Immune checkpoint molecules PD-L1 (red) and PD-1 (red) were validated on NBCL spiked into PBMC (1:20) that were stimulated for 5 days with IFN and anti-CD3/28 beads. All slides were prepared with the PFA-AC protocol. Stainings are ordered by their quality with the best on top.

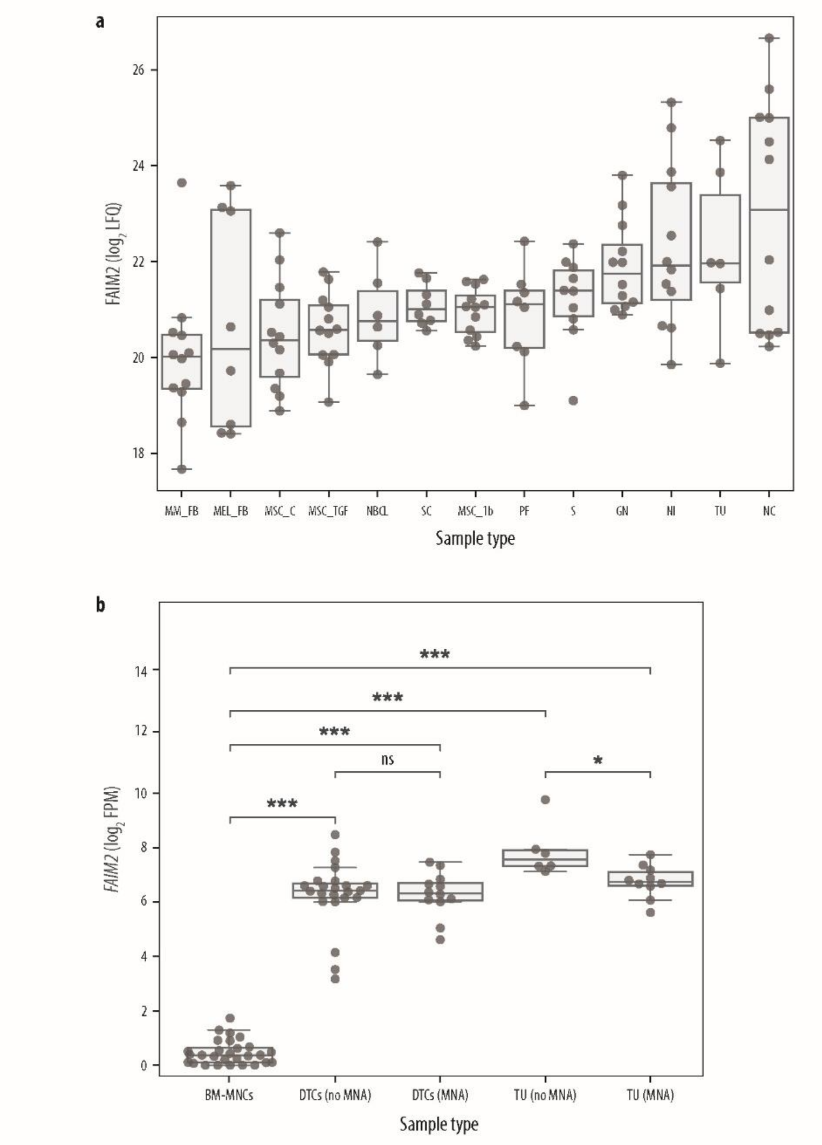

Figure S5. *FAIM2* transcription in RNA-seq data and expression in proteomics data. (**a**) *FAIM2* protein expression in fibroblasts from multiple myeloma (MM_FB), fibroblasts from melanoma (MEL_FB), mesenchymal stem cell control (MSC_C), mesenchymal stem cells TGF-β treated (MSC_TGF), neuroblastoma cells lines (NBCL), Schwann cells (SC), mesenchymal stem cells IL-β treated (MSC_1b), neuroblastoma peripheral-nerve-associated fibroblasts (PF), Schwannoma (S), Ganglioneuroma (GN), peripheral nerve fascicle induced (NI), primary tumors (TU) and peripheral nerve fascicle control (NC). LFQ, Label Free Quantification. (**b**) *FAIM2* mRNA transcription in bone marrow-derived mononuclear cells (BM-MNCs), DTCs as well as neuroblastoma primary tumors (TU) with and without *MYCN* amplification (MNA). Wilcoxon-Mann-Whitney with FDR-corrected p-values: ns, p> 0.05, *, $p \leq 0.05$; **, $p \leq 0.01$; ***, $p \leq 0.001$. FPM, fragments per million.

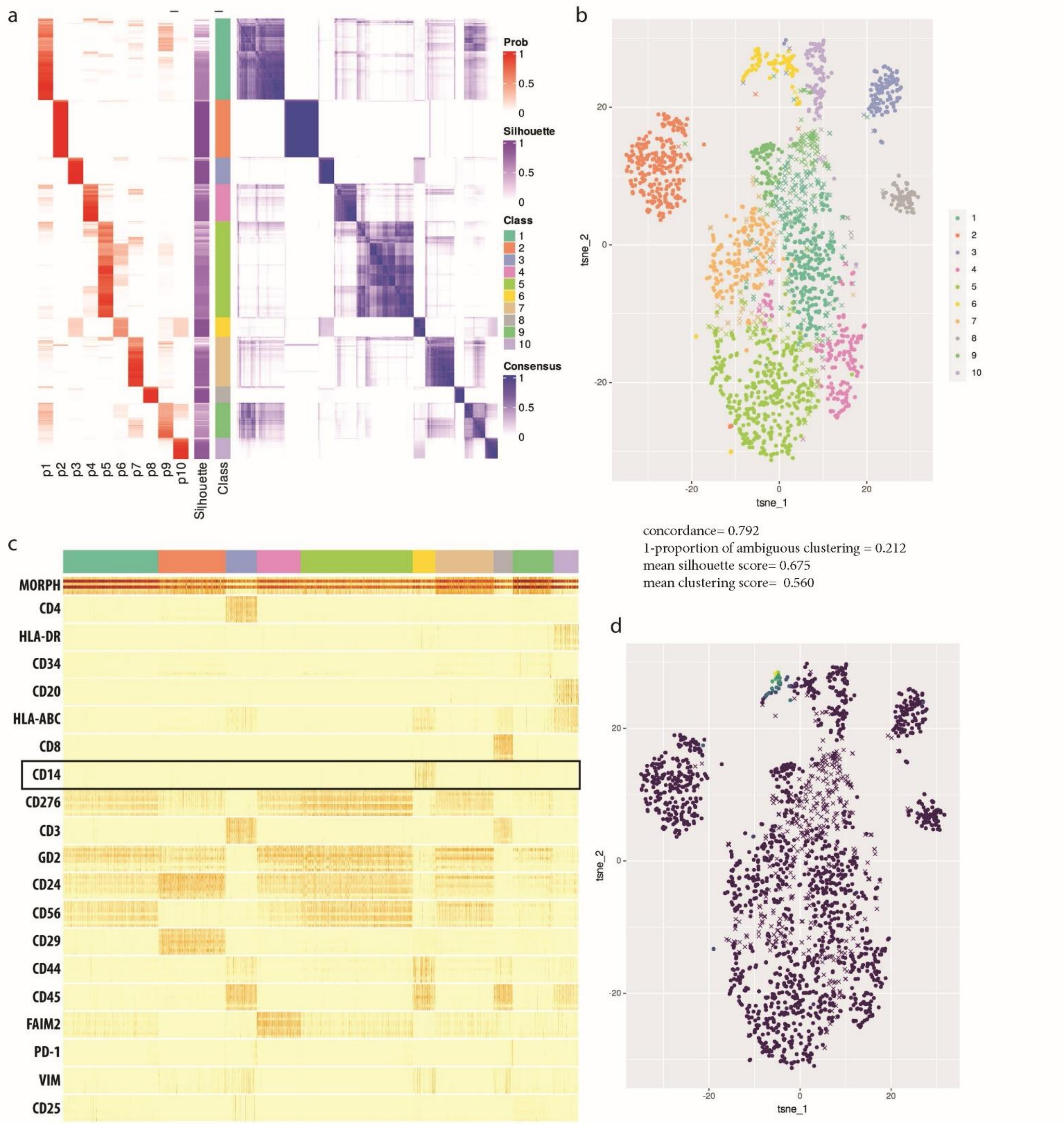

Figure S6. Cluster specificity and stability using all features. Cluster specificity and stability determined by consensus clustering on 2021 single-cell vectors comprised of *all features* from one representative BM sample (first FoV of BM 1.1) using the Bioconductor package, cola [15]. (**a**) left, probability heatmap showing probability of a cell (y-axis) appearing in a cluster (x-axis); right, consensus heatmap showing how often two cells (single cells on x- and y-axis) appear in the same cluster. Silhouette, silhouette score per cell. (**b**) tSNE showing distribution of single-cells between 10 clusters. Dots, single cells with silhouette score > 0.5; cross, single cells with silhouette score < 0.5. (**c**) Heatmap showing normalized single-cell feature values in 10 created clusters. MORPH, morphological features (from top to bottom: size nucleus, solidity nucleus, perimeter nucleus, roundness nucleus, size cell, perimeter cell); 9 intensity features per marker from top to bottom: mean intensity, total intensity and mean of the 20% highest pixel intensities in nucleus, cell and cytoplasm/membrane. (**d**) tSNE colored by CD14 signal intensity (mean of the highest 20% of pixel values in the cytoplasm/membrane). Dots, single cells with silhouette score > 0.5; cross, single cells with silhouette score < 0.5.

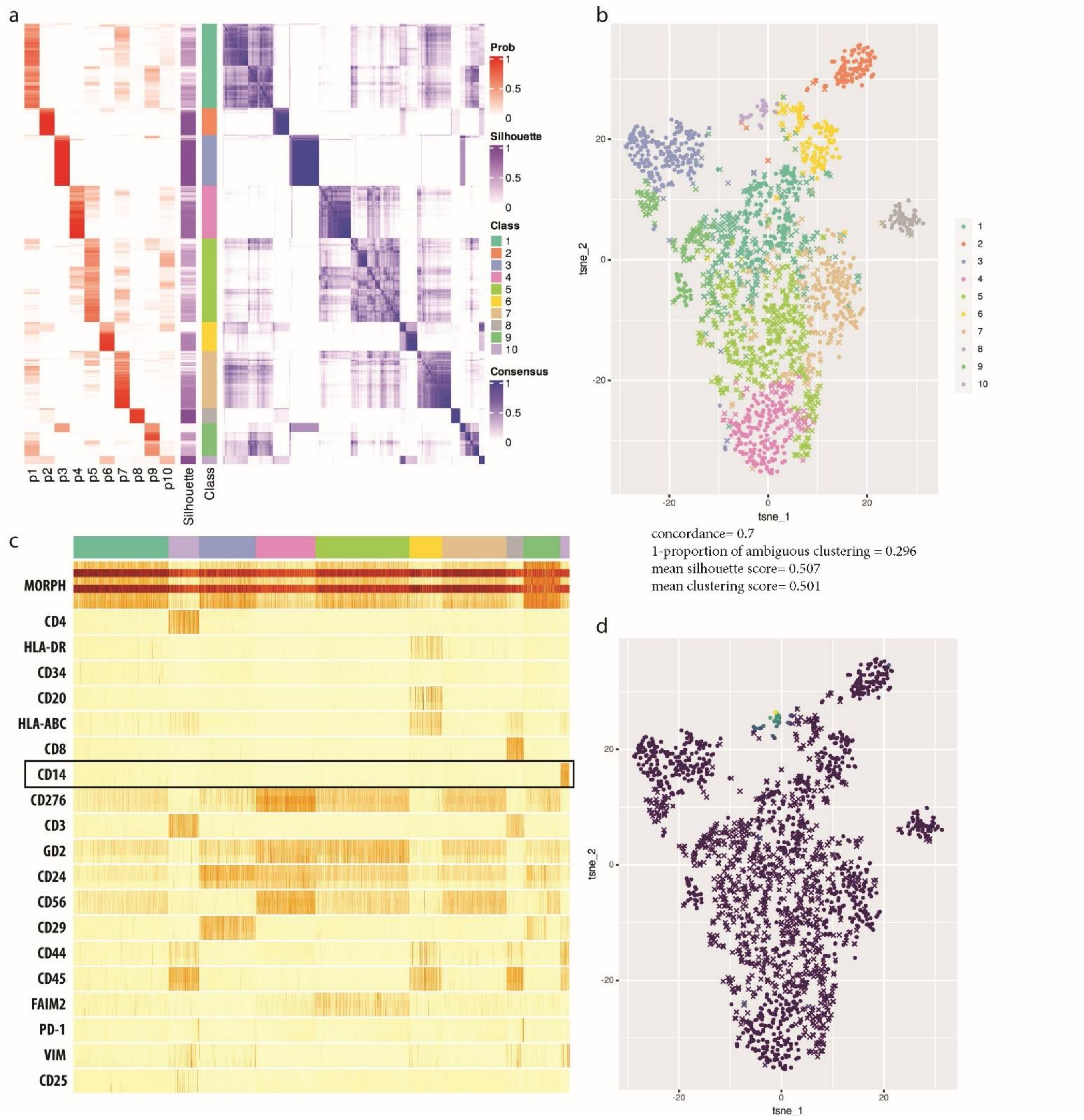

Figure S7. Cluster specificity and stability using mean of the 20% highest pixel intensities (M20) to represent marker intensity and morphological features. Cluster specificity and stability determined by consensus clustering on 2021 single-cell vectors comprised of *M20 of each marker plus morphological features* from one representative BM sample (first FoV of BM 1.1) using the Bioconductor package, cola [15]. (**a**) left, probability heatmap showing probability of a cell (y-axis) appearing in a cluster (x-axis); right, consensus heatmap showing how often two cells (single cells on x- and y-axis) appear in the same cluster. Silhouette, silhouette score per cell. (**b**) tSNE showing distribution of single-cells between 10 clusters. Dots, single cells with silhouette score > 0.5; cross, single cells with silhouette score < 0.5. (**c**) Heatmap showing normalized single-cell feature values in 10 created clusters. MORPH, morphological features (from top to bottom: size nucleus, solidity nucleus, perimeter nucleus, roundness nucleus, size cell, perimeter cell); 3 intensity features per marker from top to bottom: M20 in nucleus, cell and cytoplasm/membrane. (**d**) tSNE colored by CD14 signal intensity (M20 in the cytoplasm/membrane). Dots, single cells with silhouette score > 0.5; cross, single cells with silhouette score < 0.5.

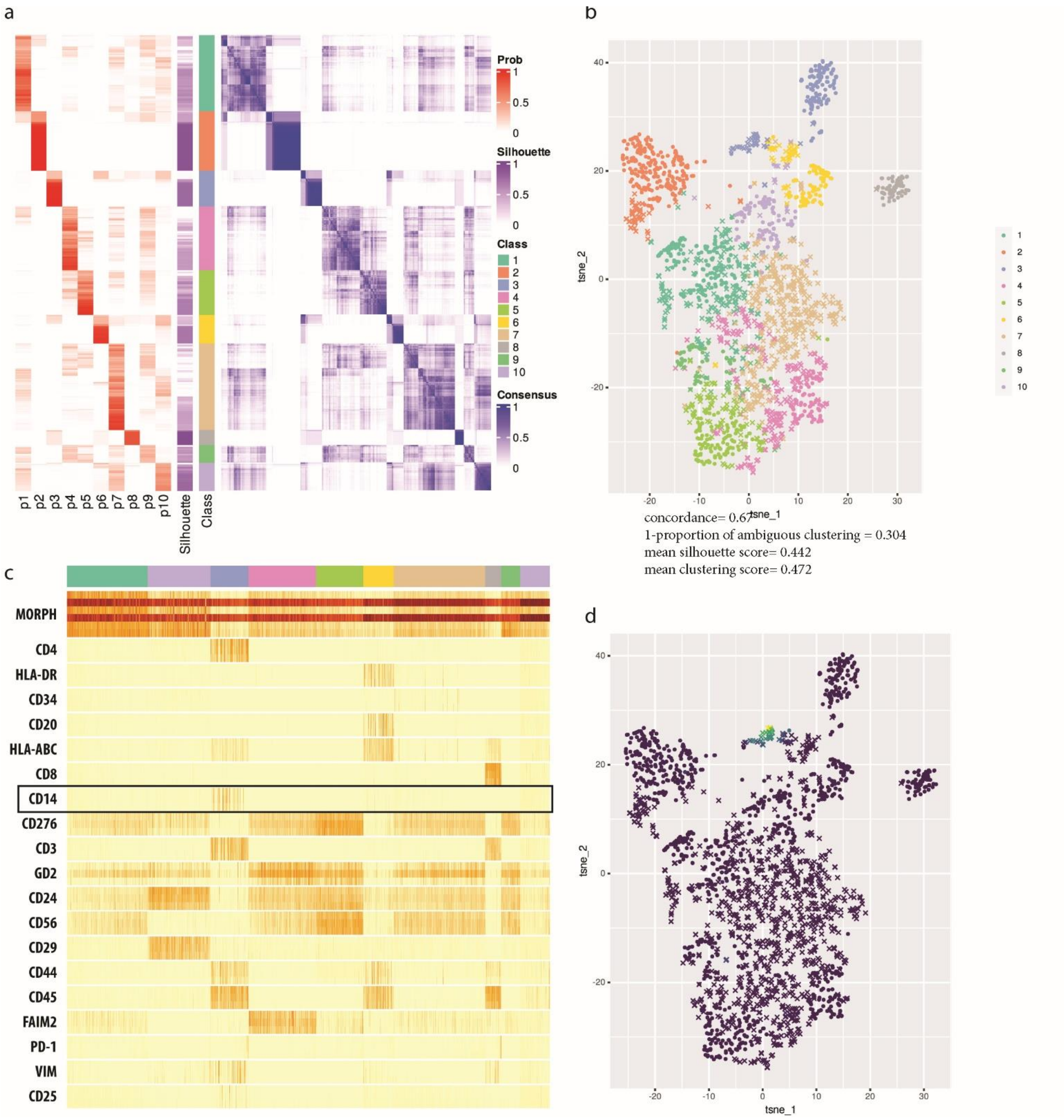

Figure S8. Cluster specificity and stability using mean intensity (ME) to represent marker intensity and morphological features. Cluster specificity and stability determined by consensus clustering on 2021 single-cell vectors comprised of *ME of each marker plus morphological features* from one representative BM sample (first FoV of BM 1.1) using the Bioconductor package, cola [15]. (**a**) left, probability heatmap showing probability of a cell (y-axis) appearing in a cluster (x-axis); right, consensus heatmap showing how often two cells (single cells on x- and y-axis) appear in the same cluster. Silhouette, silhouette score per cell. (**b**) tSNE showing distribution of single-cells between 10 clusters. Dots, single cells with silhouette score > 0.5; cross, single cells with silhouette score < 0.5. (**c**) Heatmap showing normalized single-cell feature values in 10 created clusters. MORPH, morphological features (from top to bottom: size nucleus, solidity nucleus, perimeter nucleus, roundness nucleus, size cell, perimeter cell); 3 intensity features per marker from top to bottom: ME in nucleus, cell and cytoplasm/membrane. (**d**) tSNE colored by CD14 signal intensity (ME in the cytoplasm/membrane). Dots, single cells with silhouette score > 0.5; cross, single cells with silhouette score < 0.5.

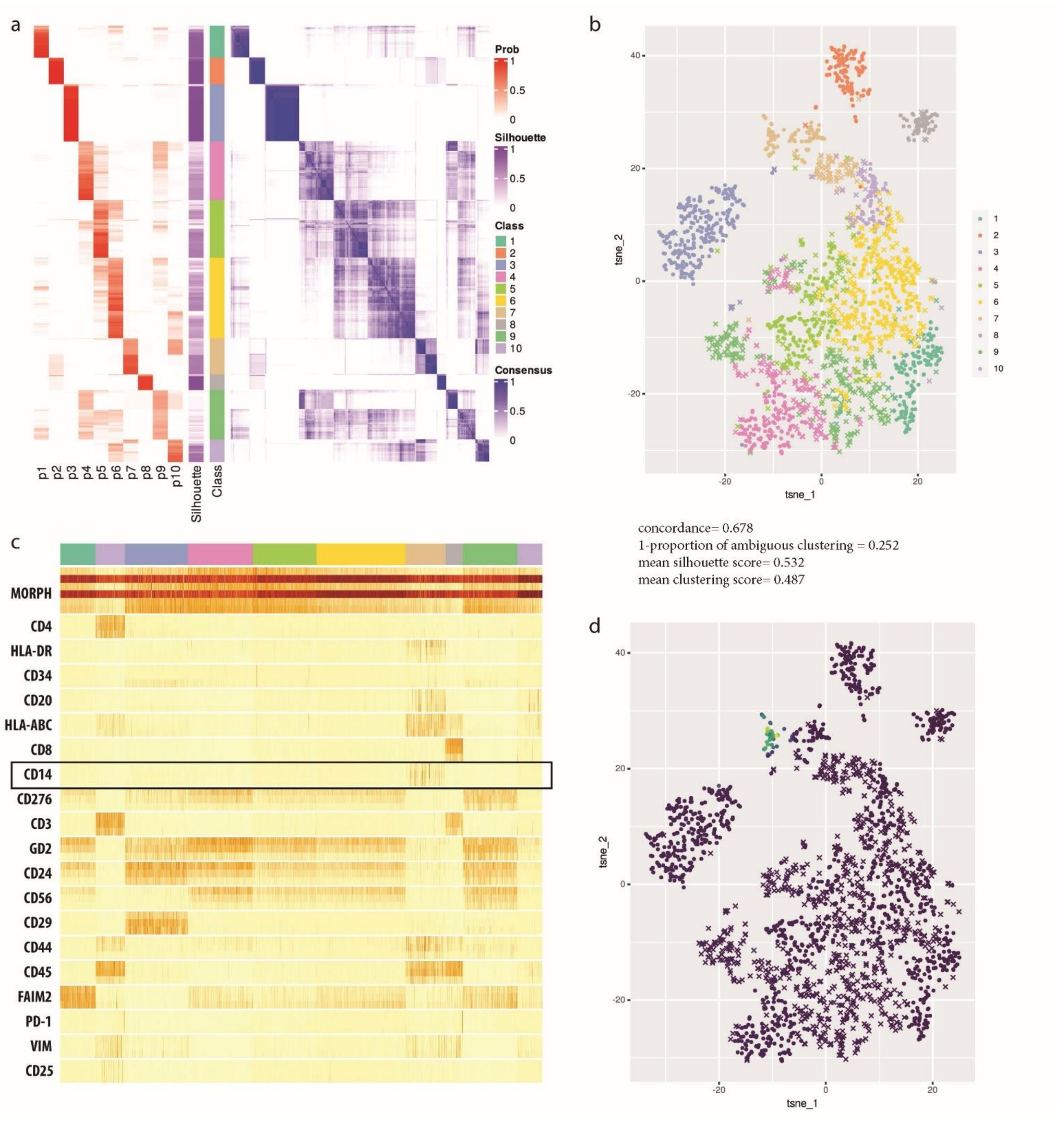

Figure S9. Cluster specificity and stability using total intensity (TO) to represent marker intensity and morphological features. Cluster specificity and stability determined by consensus clustering on 2021 single-cell vectors comprised of *TO of each marker plus morphological features* from one representative BM sample (first FoV of BM 1.1) using the Bioconductor package, cola[15]. (**a**) left, probability heatmap showing probability of a cell (y-axis) appearing in a cluster (x-axis); right, consensus heatmap showing how often two cells (single cells on x- and y-axis) appear in the same cluster. Silhouette, silhouette score per cell. (**b**) tSNE showing distribution of single-cells between 10 clusters. Dots, single cells with silhouette score > 0.5; cross, single cells with silhouette score < 0.5. (**c**) Heatmap showing normalized single-cell feature values in 10 created clusters. MORPH, morphological features (from top to bottom: size nucleus, solidity nucleus, perimeter nucleus, roundness nucleus, size cell, perimeter cell); 3 intensity features per marker from top to bottom: TO in nucleus, cell and cytoplasm/membrane. (**d**) tSNE colored by CD14 signal intensity (TO in the cytoplasm/membrane). Dots, single cells with silhouette score > 0.5; cross, single cells with silhouette score < 0.5.

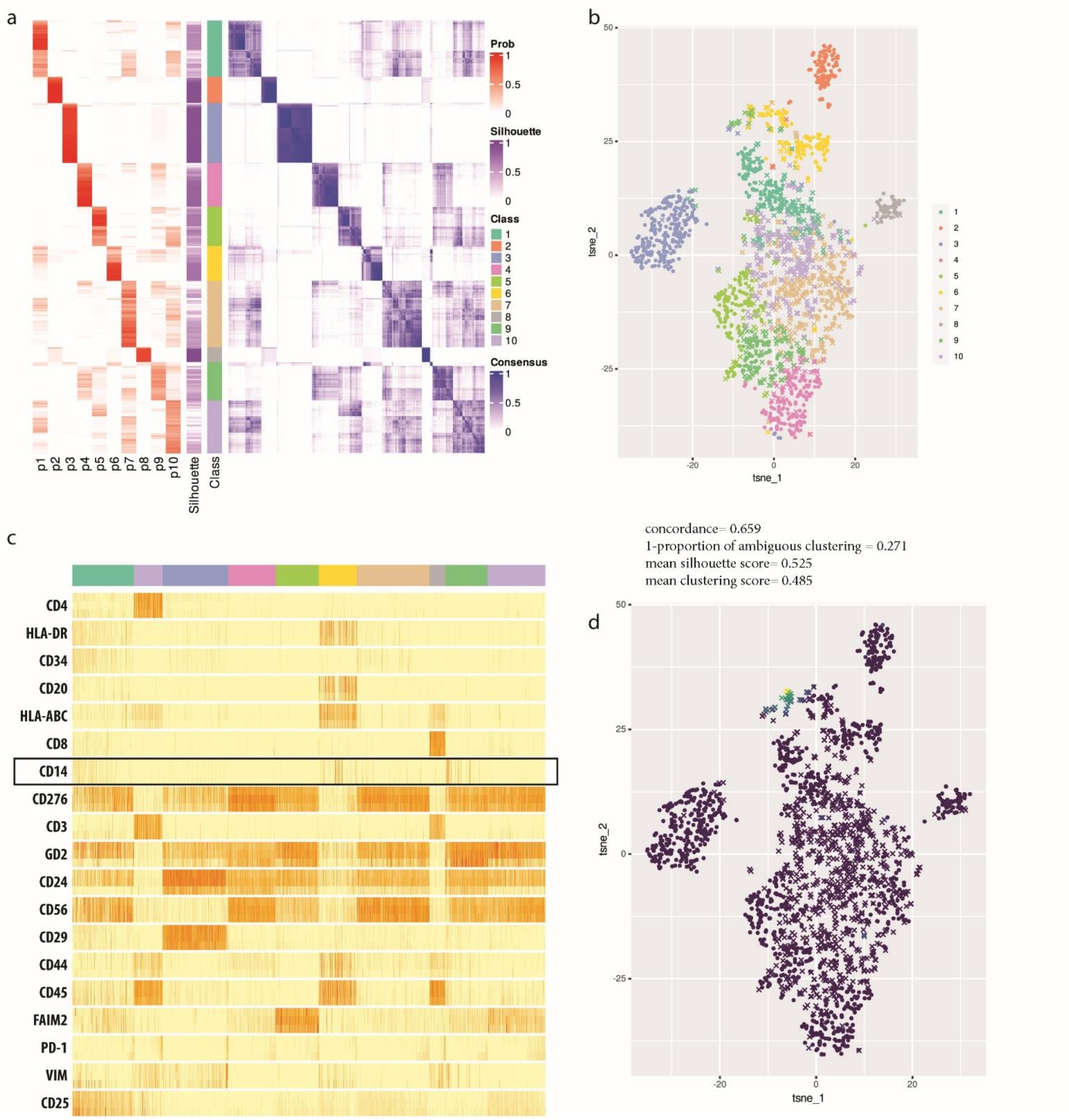

Figure S10. Cluster specificity and stability using mean of the 20% highest pixel intensities (M20) to represent marker intensity without morphological features. Cluster specificity and stability determined by consensus clustering on 2021 single-cell vectors comprised of *M20 of each marker without morphological features* from one representative BM sample (first FoV of BM 1.1) using the Bioconductor package, cola[15]. (**a**) left, probability heatmap showing probability of a cell (y-axis) appearing in a cluster (x-axis); right, consensus heatmap showing how often two cells (single cells on x- and y-axis) appear in the same cluster. Silhouette, silhouette score per cell. (**b**) tSNE showing distribution of single-cells between 10 clusters. Dots, single cells with silhouette score > 0.5; cross, single cells with silhouette score < 0.5. (**c**) Heatmap showing normalized single-cell feature values in 10 created clusters. Three intensity features per marker from top to bottom: M20 in nucleus, cell and cytoplasm/membrane. (**d**) tSNE colored by CD14 signal intensity (M20 in the cytoplasm/membrane). Dots, single cells with silhouette score > 0.5; cross, single cells with silhouette score < 0.5.

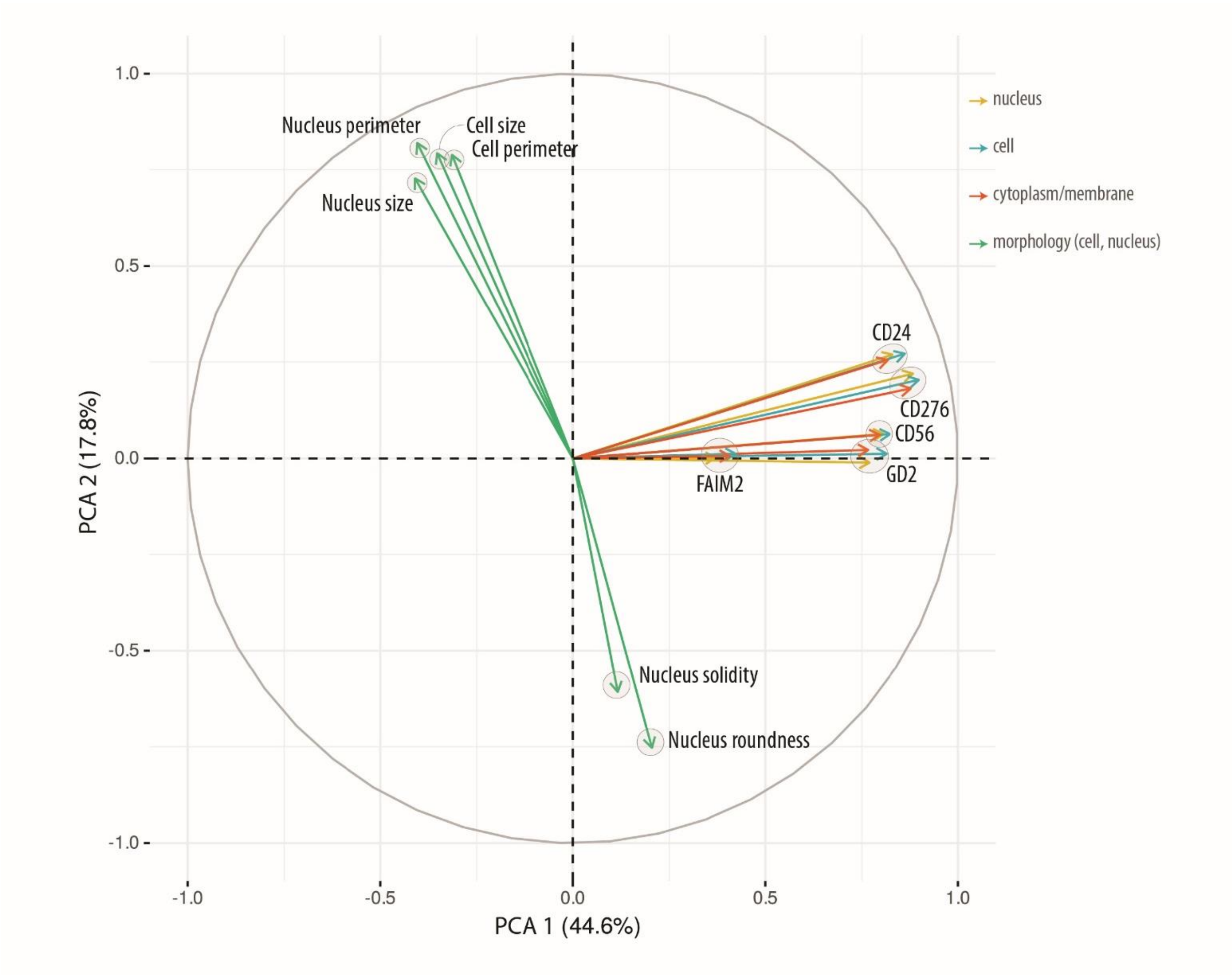

Figure S11. Contribution of morphological features to DTC heterogeneity. Biplot representation of principal component analysis (PCA) on the autoscaled data (single-cell vectors comprised of M20 of each DTC marker and morphological features) of all DTC clusters (cluster 1, 4, 5, 7 and 9) in one representative bone marrow sample (1.FoV of BM 1.1), showing the projection of the data set in the PC1xPC2 plane. Length of adjacent and opposite leg of arrows show contribution to first and second principal component, respectively. Green, morphological features; blue, M20 in cell: yellow; M20 in nucleus; red, M20 in cytoplasm/membrane.

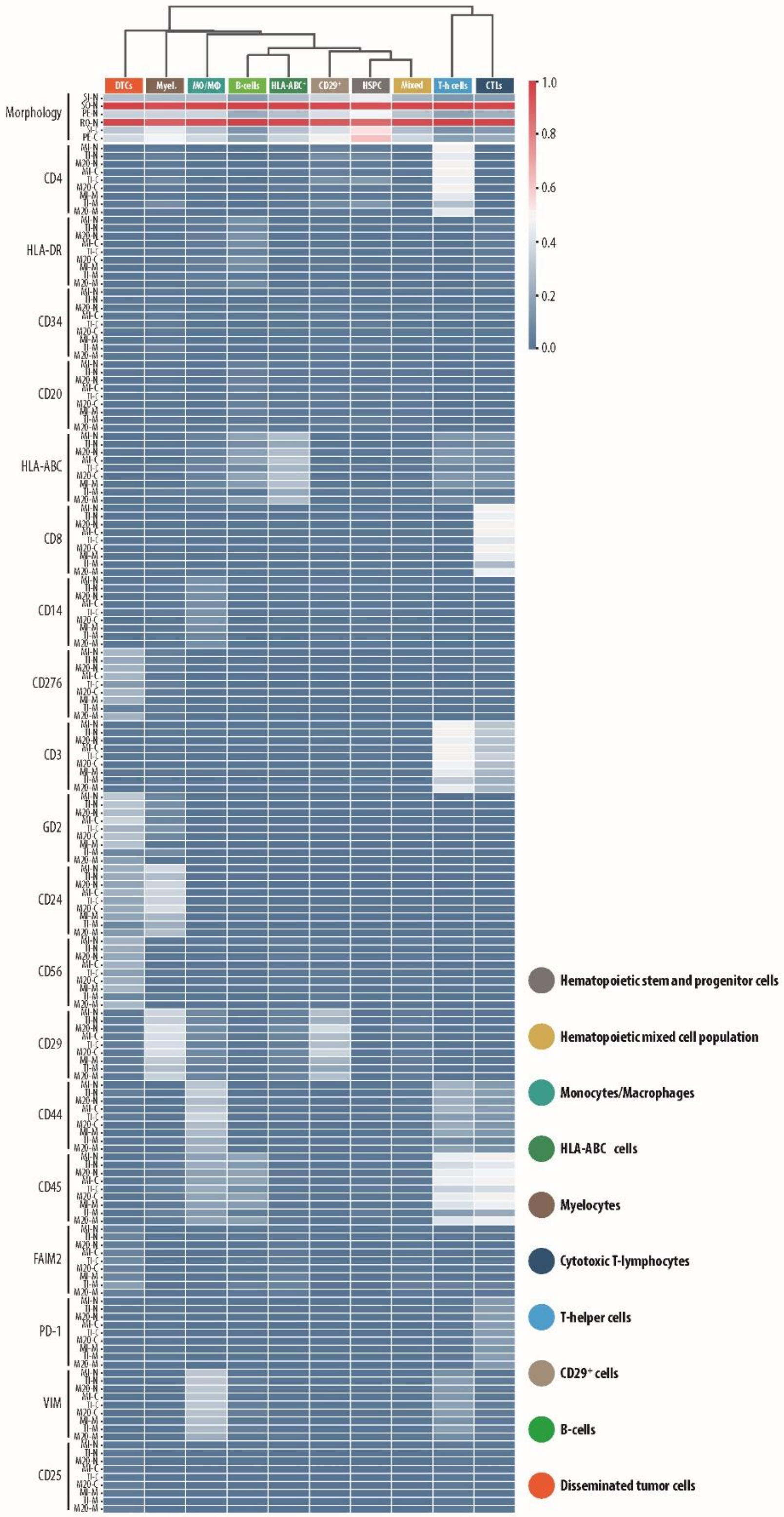

Figure S12. Heatmap of 10 cell types without feature-wise scaling. Heatmap showing the median feature values of 10 clusters (created by by A-tSNE [16] and subsequent GMS [17] in Cytosplore [14] without feature-wise scaling. SI, size; SO, solidity; PE, perimeter; RO, roundness; MI, mean intensity; TI, total intensity; M20, mean of the highest 20% of pixel values; -N, nucleus; -C, cell; -M, cytoplasm/membrane; DTCs, disseminated tumor cells; Myel., myelocytes; MO/MΦ, monocytes/macrophages; HSPC, hematopoietic stem and progenitor cells; T-h cells, T-helper cells; CTLs; cytotoxic T-lymphocytes; Mixed, hematopoietic mixed cell population.

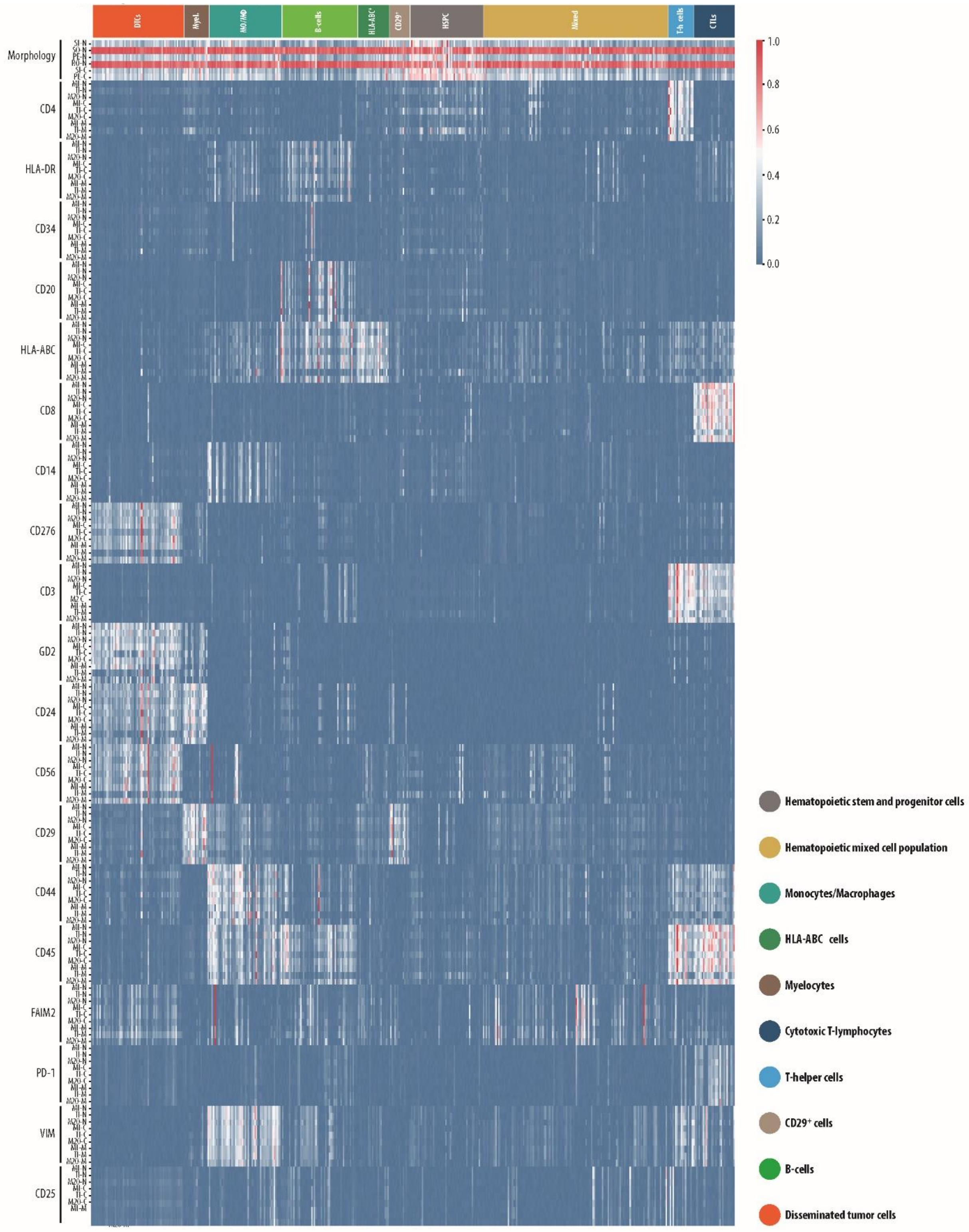

Figure S13. Heatmap of single-cell values of 10 A-tSNE clusters. Heatmap showing normalized single-cell feature values of 10 created clusters (created by by A-tSNE and subsequent GMS [17] in Cytosplore [14].SI, size; SO, solidity; PE, perimeter; RO, roundness; MI, mean intensity; TI, total intensity; M20, mean of the highest 20% of pixel values; -N, nucleus; -C, cell; -M, cytoplasm/membrane; DTCs, disseminated tumor cells; Myel., myelocytes; MO/MΦ, monocytes/macrophages; HSPC, hematopoietic stem and progenitor cells; T-h cells, T-helper cells; CTLs; cytotoxic T-lymphocytes; Mixed, hematopoietic mixed cell population.

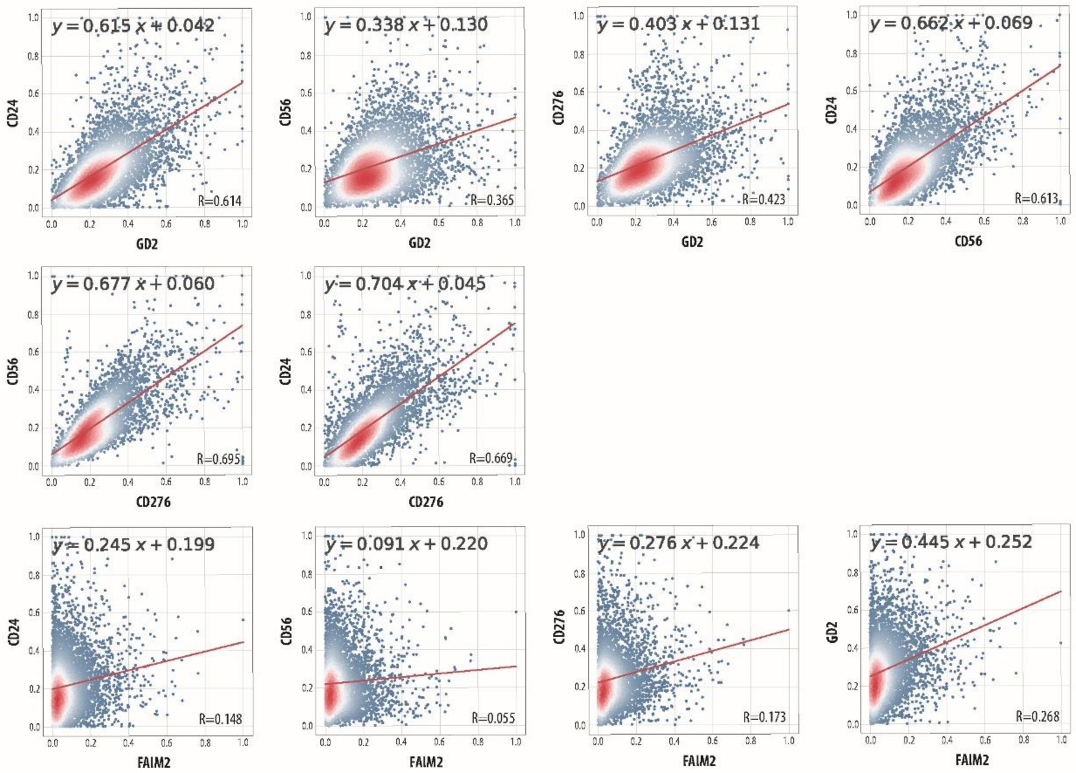

Figure S14. Correlation of DTC markers. Scatter plots showing correlations between DTC markers for all cells of the DTC cluster. Mean of the highest 20% of pixel values in the cytoplasm/membrane was used as measure for marker abundance. R, correlation coefficient; red line, linear regression.

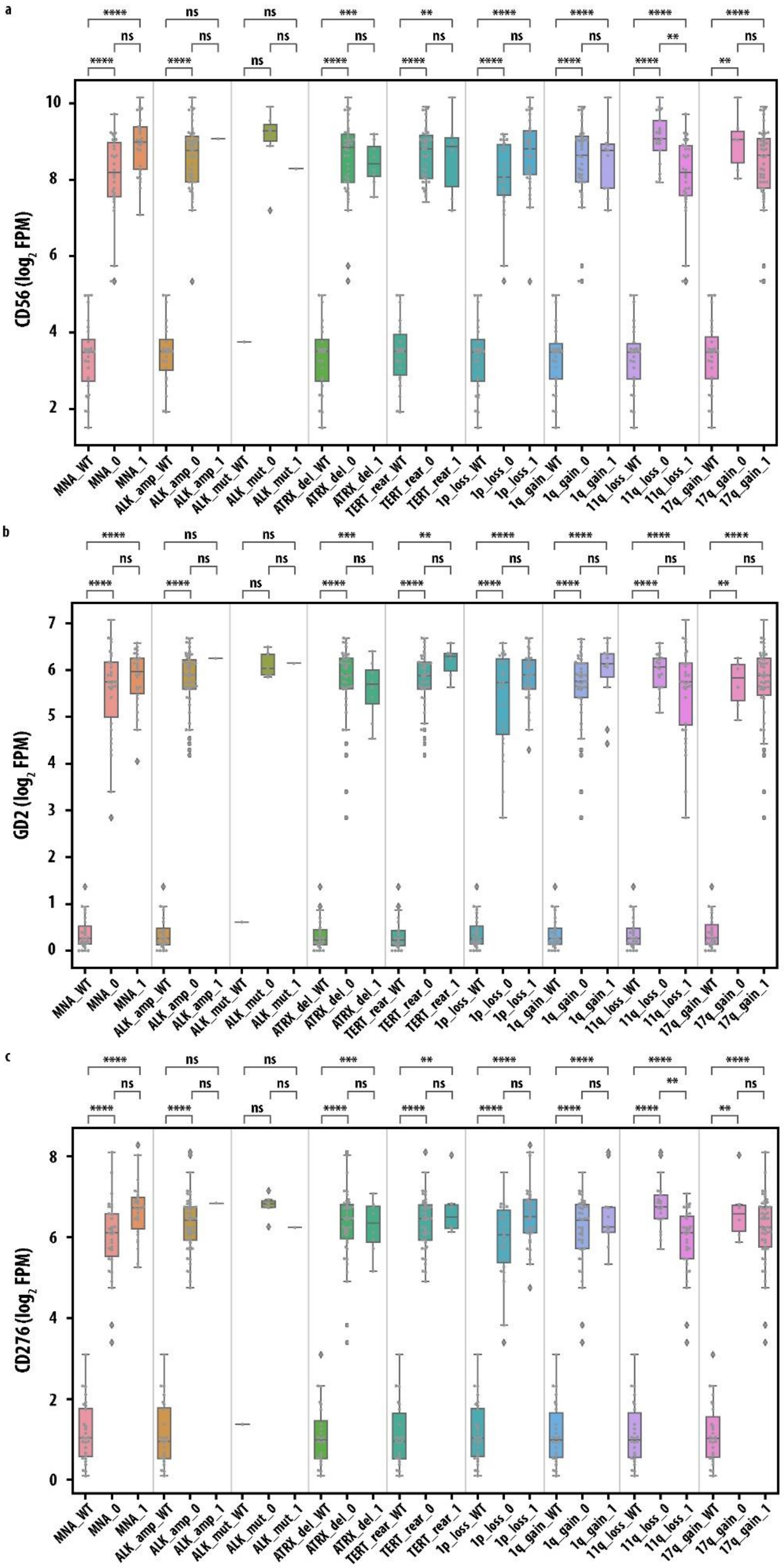
a
CD56 (log2 FPM)
b
GD2 (log2 FPM)
c
CD276 (log2 FPM)
MNA_WT
MNA_0
MNA_1
ALK_amp_WT
ALK_amp_0
ALK_amp_1
ALK_mut_WT
ALK_mut_0
ALK_mut_1
ATRX_del_WT
ATRX_del_0
ATRX_del_1
TERT_rear_WT
TERT_rear_0
TERT_rear_1
1p_loss_WT
1p_loss_0
1p_loss_1
1q_gain_WT
1q_gain_0
1q_gain_1
11q_loss_WT
11q_loss_0
11q_loss_1
17q_gain_WT
17q_gain_0
17q_gain_1

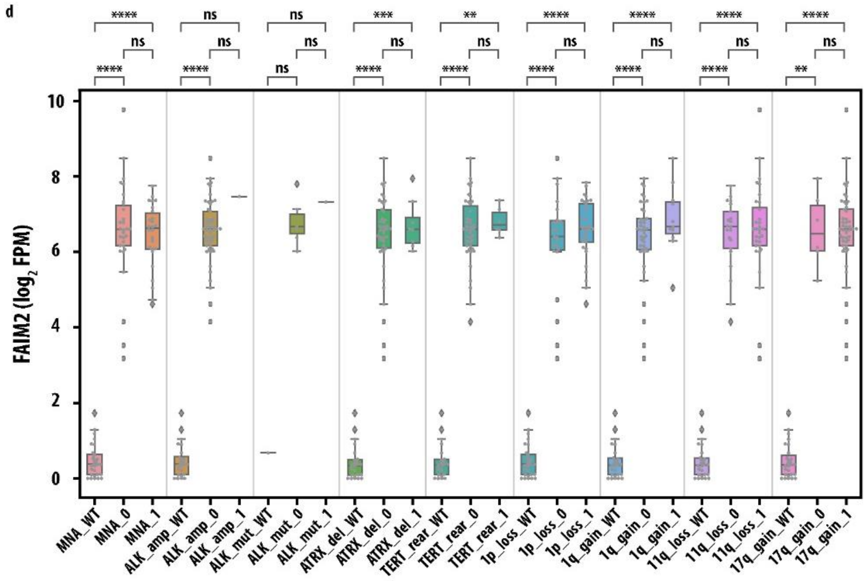

Figure S15. Correlation of DTC markers with neuroblastoma hallmark drivers. (**a–d**) *CD56, GD2, CD276 and FAIM2* mRNA transcription in bone marrow-derived mononuclear cells (wild type, WT) and neuroblastoma tumor cells without (0) and with (1) genetic aberration. MNA, *MYCN* amplification; amp, amplification; mut, mutation; rear, rearrangement. Wilcoxon-Mann-Whitney with FDR-corrected *p*-values: ns, $p > 0.05$; *, $p \leq 0.05$; **, $p \leq 0.01$; ***, $p \leq 0.001$; ****, $p \leq 0.0001$. FPM, fragments per million.

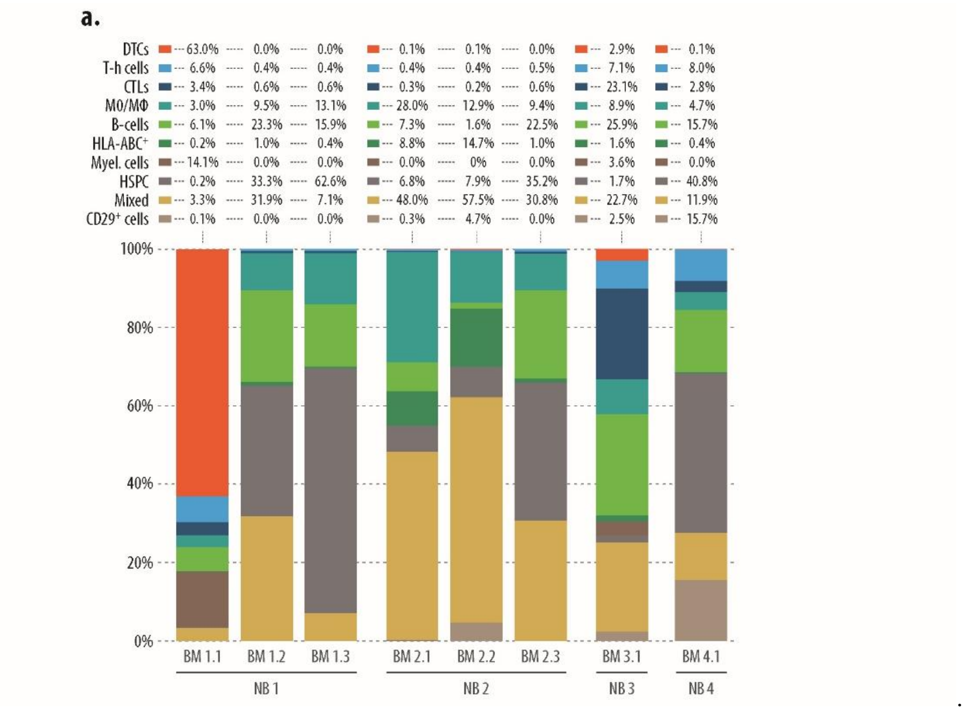

.

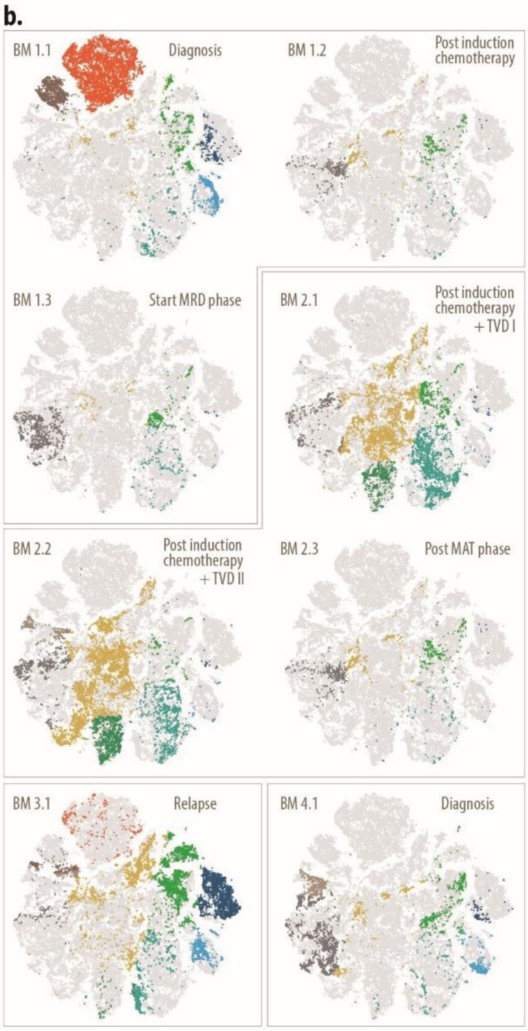

Figure S16. Cell composition in each of the eight bone marrow samples. (**a**) Bar charts demonstrating the cell composition of 8 analyzed bone marrow samples. DTCs, disseminated tumor cells; Myel., myelocytes; MO/MΦ, monocytes/macrophages; Mes. cells, mesenchymal cells; HSPC, hematopoietic stem and progenitor cells; T-h cells, T-helper cells; CTLs; cytotoxic T-lymphocytes; Mixed, hematopoietic mixed cell population. (**b**) Cell type distribution in each of the 8 BM samples. TVD, topotecan-vincristine-doxorubicin; MAT, myeloablative therapy with autologous stem cell transplantation; MRD, minimal residual disease.

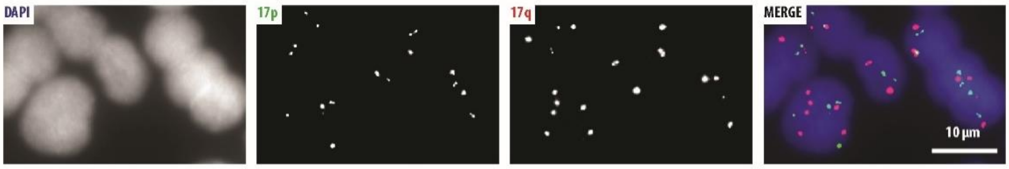

Figure S17. FISH analysis on a sample of lymph node metastases. FISH analysis with chromosome 17-specific probe on sample of lymph node metastases collected from neuroblastoma patient NB1 with 17pq gain. 4 copies of 17p (green) and 6 copies of 17q (red) were detected on DTCs. Nuclei were counterstained with DAPI (blue).

3. Tables

Table S1. 5 Neuroblastoma cell lines (NBCL) used for biomarker validation. BM, bone marrow; DTCs, disseminated tumor cells; INRG; International Neuroblastoma Risk Group.

NBCL	Derived From	INRG Stage	*MYCN* Amplification
STA-NB-2	Primary tumor	M	no
STA-NB-4	Primary tumor	M	yes
STA-NB-8	BM-derived DTCs	M	yes
STA-NB-10	Primary tumor	L2	yes
STA-NB-12	BM-derived DTCs	M	no

Table S2. Patient and sample set comprised of eight bone marrow samples. INRG, International Neuroblastoma Risk Group Staging System, HRNBL1, High Risk Neuroblastoma study 1; LINES, Low and Intermediate Risk Neuroblastoma European Study; MNA, *MYCN* amplification; neg, negative; pos, positive; TVD, topotecan-vincristine-doxorubicin; MAT, myeloablative therapy with autologous stem cell transplantation. Tumor cell content was detected by DeepFLEX.

Patient ID	*Sex*	*Age at Diagnosis (Months)*	*INRG*	*Clinical Study*	*Event*	*Dead/Alive*	*Sample ID*	*Time Point During Therapy*	*DeepFLEX: Absolute Number of DTCs*	*Total Number of Cells Analyzed*
NB1	F	54	M	HRNBL1	no	alive	BM1.1	diagnosis	4,827	7,672
							BM1.2	post induction chemotherapy	0	841
							BM1.3	start MRD phase	0	1,381
NB2	M	8.5	M	no	no	alive	BM2.1	post induction + TVD	3	6,044
							BM2.2	post induction + TVD II	8	7,474
							BM2.3	post MAT	0	1,020
NB3	F	86	M	HRNBL1	yes	dead	BM3.1	relapse	223	7,788
NB4	F	5	Ms	LINES	no	alive	BM4.1	diagnosis	2	3,480

Table S8. Parameters used for CIDRE and Mask R-CNN in DeepFLEX.

	Parameter	Value
CIDRE	Illumination gain regularization (lambda_v)	0.17
	Zero-light regularization (lamba_z)	0.1
	Correction mode (correction_mode)	0 (zero-light preserved)
	Q-ratio (q_percent)	0.25
	Bit-depth (bit-depth)	16 bit
	Max. iterations (max_lbfgs_iterations)	10, 000
Mask R-CNN	Data augmentation	Flip left/right, up/down, artificially synthesized images
	Optimizer	Stochastic gradient descent
	Epoches	300

Table S9. Morphological and intensity features to describe single cells.

	Feature	Description
Morphology	size	sum of pixels of an object
	perimeter	sum of pixels around object boundary, calculated using the python package scikit-image (scikit-image.org/)
	roundess (r)	$r = \frac{4\pi \cdot area}{perimeter^2}$, where area was computed using the python package scikit-image
	solidity (s)	$s = \frac{area}{area(convexhull)}$, where convex hull was computed using the python package scikit-image
Intensity	total intensity	sum of pixel intensities of an object
	mean intensity	mean of pixel intensities of an object
	mean of the highest 20% of all pixel intensities	mean of the highest 20% of pixel intensities of an object

Table S12. Three centrifugation and fixation methods. Pre-fixation: Slides together with their cyto chambers were taken out of the centrifuge. After aspiration, 100µL of 4°C-cold, 4% PFA were carefully applied onto the slide along the wall of the funnel chamber. Fixation was carried out at 4°C by leaving the cyto chamber with the inserted slide in the fridge for 90 s. Filter paper and funnel chamber were removed after the second aspiration cycle in all three methods.

Step	Acetone (AC)	PFA	PFA plus Acetone (PFA-AC)
1st centrifugation	1000 g, 8 min	1000 g, 8 min	1000 g, 8 min
aspiration	yes	yes	yes
pre-fixation	-	-	4% PFA in PBS, 90 s, 4°C
aspiration	-	-	yes
2nd centrifugation	1300 g, 4 min	1300 g, 4 min	1300 g, 4 min
drying	1 h 30 min, RT	1 h 30 min, RT	1 h 30 min, RT
fixation	Acetone, 20 s, RT	4% PFA in PBS, 24 h, 4°C	Acetone, 20 s, RT

Table S13. Acetone and PFA scores. Evaluation of IF-stainings of selected biomarkers on 5 distinct neuroblastoma cell line (NBCL) samples prepared with the corresponding protocol.

Antigen	AC Overall	PFA Overall	AC Morphology	PFA Morphology	AC Staining	PFA Staining

GD2	21.2	20.8	8.4	9	12.8	11.8
CD56	22.4	19	8.3	8.6	14.1	10.4
TAG1	18.9	19.4	8	8.9	10.9	10.5
DCLK1	17.3	13.1	7.7	7.9	9.6	5.2
FAIM2	21.5	18.5	9.1	9.1	12.4	9.4
PRAME	17.3	12.5	8.2	7.75	9.1	4.75
TACC2	11	12	7	8	4	4
NCAM-L1	18.7	16.9	8.3	9.6	10.4	7.3
VIM	17.7	16.2	7.5	8.8	10.2	7.4
PROM1	17.8	12.8	8.2	8.8	9.6	4
B7-H3	19.5	13.4	8.3	9	11.2	4.4

Table S14. All primary and secondary antibodies tested in present study.

Antibody	Conju-gate	Class∣Host∣Isotype	Clone	Supplier	Catalogue-Number	Optimal Dilution
**B7-H3**	PE	human IgG1	REA1094	Miltenyi Biotec	130-118-570	1:40
CD14	PE	monoclonal mouse IgG	OFC14D	ImmunoTools	21450144	1:20
**CD14**	PE	monoclonal mouse IgG1	18D11	ImmunoTools	21620144	1:20
CD20	PE	monoclonal mouse IgG2a	LT20	ImmunoTools	21279204	1:20
**CD20**	PE	recombinant human IgG1	REA780	Miltenyi Biotec	130-111-338	1:20
**CD24**	FITC	monoclonal mouse IgG1	SN3	ImmunoTools	21270243	1:20
**CD25**	PE	monoclonal mouse IgG	HI25a	ImmunoTools	21810254	1:20
**CD29**	FITC	monoclonal mouse IgG1	HI29a	ImmunoTools	21810293	1:20
CD3	FITC	monoclonal mouse IgG1	HIT3b	ImmunoTools	21810033	1:20
**CD3**	PE	monoclonal mouse IgG1	UCHT1	ImmunoTools	21620034	1:20
**CD34**	PE	monoclonal mouse IgG1	4H11[APG]	ImmunoTools	21270344	1:20
CD4	PE	monoclonal mouse IgG1k	IT4	ImmunoTools	21459044	1:20
**CD4**	PE	monoclonal mouse IgG2a,k	VIT4	Miltenyi Biotec	130-113-214	1:20

**CD44**	PE	monoclonal rat IgG2b	IM7	ImmunoTools	21850444	1:20
**CD45**	PE	monoclonal mouse IgG1	HI30	ImmunoTools	21810454	1:20
**CD56**	PE	monoclonal mouse IgG1	B-A19	ImmunoTools	21810564S	1:20
**CD8**	PE	monoclonal mouse IgG1	HIT8a	ImmunoTools	21810084	1:20
DCLK1	unconj.	polyclonal rabbit IgG		Abcam	ab31704	1:50
**FAIM2**	unconj.	polyclonal rabbit IgG		ThermoFisher	PA5-20311	1:50
**GD2**	FITC	ch14.18/deltaCH2 (hamster/human)		Tuebingen		1:100
**HLA-ABC**	PE	monoclonal mouse IgG2a	W6/32	ImmunoTools	21159034	1:20
**HLA-DR**	PE	monoclonal mouse IgG1	HI43	ImmunoTools	21819984	1:20
NCAM-L1	biotinylated	recombinant human IgG1		Miltenyi Biotec	130-100-702	1:25
**PD-1**	biotinylated	monoclonal mouse IgG1	NAT105	BioLegend	367418	1:50
PD-L1/CD274	unconj.	monoclonal mouse IgG1	22C3	Dako	M3653	1:20
**Propidium Iodide**				Genaxxon bioscience	M3181.0010	1:1000
PRAME	unconj.	monoclonal rabbit IgG	EPR20330	Abcam	ab219650	1:25
PROM1	biotinylated	monoclonal mouse IgG1	AC133	Miltenyi Biotec	130-113-107	1:25
TACC2	unconj.	polyclonal rabbit IgG		Abcam	ab204891	1:10
TAG1	unconj.	monoclonal rabbit IgG	EPR5106	Abcam	ab133498	1:50
Vimentin	unconj.	recombinant chicken IgY		Milipore/Chemicon	AB5733	1:100
Vimentin	unconj.	monoclonal mouse IgG1	V9	Dako	M0725	1:50
Gt a Mouse	TRITC	polyclonal goat IgG		Jackson ImmunoResearch	115-025-072	1:200
Gt α Ch	FITC	polyclonal goat IgG		ThermoFisher	A16055	1:500
Ms α Biot.	Cy3	monoclonal mouse IgG	3D6.6	Jackson ImmunoResearch	200-162-211	1:800
Sw α Rb	FITC	polyclonal swine IgG		Dako	F0205	1:50

Bold: Primary antibodies, which passed the validation procedure (see Methods).

Table S15. Mutually exclusive marker pairs. Mutually exclusive marker pairs based on biologically-known information (left panel) and using a data-driven approach based on singular value decomposition (right panel). Green color indicates that matched pairs exist in both panels.

**Approach**	**Knowledge-Based Approach**					**Data-Driven Approach**					**To Use for Re-store**
**Ref. marker**	**1st**	**2nd**	**3rd**	**4th**	**5th**	**1st**	**2nd**	**3rd**	**4th**	**5th**	**Final marker**
CD14	CD3	CD4	CD8			PD-1	CD24	CD8	CD3	CD20	CD3
CD20	CD14	CD3	CD4	CD8		PD-1	CD4	CD276	CD56	CD3	CD3
CD24	CD3	CD4	CD8			CD45	CD8	CD25	CD3	CD14	CD3
CD25	CD14					CD24	FAIM2	CD29	CD276	CD34	CD14
CD276	CD34	CD14				PD-1	Vimentin	HLA-ABC	CD45	CD25	CD14
CD29	CD3					CD45	PD-1	CD8	Vimentin	CD25	CD3
CD3	CD14	CD20	CD34			CD24	CD29	CD34	HLA-DR	CD20	CD20
CD34	CD4	CD8	CD14			CD25	CD8	CD45	CD3	GD2	CD8
CD4	CD14	CD8	CD20	CD34		CD29	CD8	CD20	CD56	GD2	CD8
CD44	CD3	CD4	CD20			PD-1	CD29	CD24	CD20	CD25	CD20
CD45	GD2	CD276	CD34			CD24	CD29	CD276	CD56	CD34	CD34
CD56	CD14	CD34				Vimentin	PD-1	HLA-ABC	CD45	HLA-DR	CD14
CD8	CD14	CD4	CD20	CD34		CD24	CD29	FAIM2	CD4	CD276	CD4
FAIM2	CD3	CD20	CD34	CD14		PD-1	CD25	CD8	CD20	CD45	CD20
GD2	CD4	CD8	CD14	CD45		Vimentin	HLA-ABC	PD-1	HLA-DR	CD45	CD45
HLA-ABC	GD2	CD276				GD2	CD56	CD276	CD29	CD25	GD2
HLA-DR	GD2	CD276				GD2	PD-1	CD3	CD56	CD276	GD2
PD-1	CD276	GD2				FAIM2	CD276	GD2	CD56	CD29	CD276
Vimentin	CD3	CD4	CD8			GD2	CD56	CD276	CD29	CD25	CD8

Table S16. Feature validation. Cluster stability and specificity, using different combinations of features, evaluated by three metrics: proportion of ambiguous clustering (PAG), mean silhouette score (MSS), concordance (CON), and their mean. MCS, mean clustering score; All, all features; M20 with MF, mean of the highest 20% of pixel values of each marker with morphological features; ME with MF, mean intensity of each marker with morphological features; TO with MF, total intensity of each marker with morphological features; M20 w/o MF, mean of the highest 20% of pixel values of each marker without morphological features.

Feature	1-PAC	MSS	CON	MCS
All	0.212	0.675	0.792	0.560
M20 with MF	0.296	0.507	0.7	0.501
ME with MF	0.304	0.442	0.67	0.472
TO with MF	0.252	0.532	0.678	0.487
M20 w/o MF	0.271	0.525	0.659	0.485

References

1. Rifatbegovic, F.; Frech, C.; Abbasi, M.R.; Taschner-Mandl, S.; Weiss, T.; Schmidt, W.M.; Schmidt, I.; Ladenstein, R.; Ambros, I.M.; Ambros, P.F. Neuroblastoma cells undergo transcriptomic alterations upon dissemination into the bone marrow and subsequent tumor progression. *Int. J. Cancer* **2018**, *142*, 297–307, doi:10.1002/ijc.31053.
2. Love, M.I.; Huber, W.; Anders, S. Moderated estimation of fold change and dispersion for RNA-seq data with DESeq2. *Genome Biol.* **2014**, *15*, 1–21, doi:10.1186/s13059-014-0550-8.
3. Consortium, T.U. UniProt: a worldwide hub of protein knowledge. Nucleic Acids Res. **2018**, 47, D506-515, Available online: https://doi.org/10.1093/nar/gky1049.
4. Uhlén, M.; Fagerberg, L.; Hallström, B.M.; Lindskog, C.; Oksvold, P.; Mardinoglu, A.; Sivertsson, Å.; Kampf, C.; Sjöstedt, E.; Asplund, A.; et al. Tissue-based map of the human proteome. *Science* **2015**, *347*, 1260419, doi:10.1126/science.1260419.
5. Weiss, T.; Taschner-Mandl, S.; Bileck, A.; Slany, A.; Kromp, F.; Rifatbegovic, F.; Frech, C.; Windhager, R.; Kitzinger, H.; Tzou, C.H.; et al. Proteomics and transcriptomics of peripheral nerve tissue and cells unravel new aspects of the human Schwann cell repair phenotype. *Glia* **2016**, *64*, 2133–2153, doi:10.1002/glia.23045.
6. Beiske, K.; Ambros, P.F.; Burchill, S.A.; Cheung, I.Y.; Swerts, K. Detecting minimal residual disease in neuroblastoma patients-the present state of the art. *Cancer Lett.* **2005**, *228*, 229–240, doi:10.1016/j.canlet.2005.02.053.
7. Castriconi, R.; Dondero, A.; Augugliaro, R.; Cantoni, C.; Carnemolla, B.; Sementa, A.R.; Negri, F.; Conte, R.; Corrias, M. V.; Moretta, L.; et al. Identification of 4Ig-B7-H3 as a neuroblastoma-associated molecule that exerts a protective role from an NK cell-mediated lysis. *Proc. Natl. Acad. Sci.* **2004**, *101*, 12640–12645, doi:10.1073/pnas.0405025101.
8. Van Groningen, T.; Koster, J.; Valentijn, L.J.; Zwijnenburg, D.A.; Akogul, N.; Hasselt, N.E.; Broekmans, M.; Haneveld, F.; Nowakowska, N.E.; Bras, J.; et al. Neuroblastoma is composed of two super-enhancer-associated differentiation states. *Nat. Genet.* **2017**, *49*, 1261–1266, doi:10.1038/ng.3899.
9. Künkele, A.; Taraseviciute, A.; Finn, L.S.; Johnson, A.J.; Berger, C.; Finney, O.; Chang, C.A.; Rolczynski, L.S.; Brown, C.; Mgebroff, S.; et al. Preclinical assessment of CD171-directed CAR T-cell adoptive therapy for childhood neuroblastoma: CE7 epitope target safety and product manufacturing feasibility. *Clin. Cancer Res.* **2017**, *23*, 466–477, doi:10.1158/1078-0432.CCR-16-0354.
10. Stock, C.; Ambros, I.; Lion, T. Detection of numeric and structural chromosome abnormalities in pediatric germ cell tumors by means of interphase cytogenetics. *Genes Chrom. Cancer* **1994**, *11*, 40–50.
11. Chang, Y.H.; Chin, K.; Thibault, G.; Eng, J.; Burlingame, E.; Gray, J.W. RESTORE: Robust intEnSiTy nORmalization mEthod for multiplexed imaging. *Commun. Biol.* **2020**, *3*, 1–9, doi:10.1038/s42003-020-0828-1.
12. Smith, K.; Li, Y.; Piccinini, F.; Csucs, G.; Balazs, C.; Bevilacqua, A.; Horvath, P. CIDRE: An illumination-correction method for optical microscopy. *Nat. Methods* **2015**, *12*, 404–406, doi:10.1038/nmeth.3323.
13. Kromp, F.; Fischer, L.; Bozsaky, E.; Ambros, I.M.; Dorr, W.; Beiske, K.; Ambros, P.F.; Hanbury, A.; Taschner-Mandl, S. Evaluation of Deep Learning architectures for complex immunofluorescence nuclear image segmentation. *IEEE Trans. Med. Imaging* **2021**, doi:10.1109/TMI.2021.3069558.
14. Waskom, M.; Botvinnik, O.; Gelbart, M.; Ostblom, J.; Hobson, P.; Lukauskas, S.; Gemperline, D.C.; Augspurger, T.; Halchenko, Y.; Warmenhoven, J.; et al. Seaborn 2020.
15. Höllt, T.; Pezzotti, N.; van Unen, V.; Koning, F.; Eisemann, E.; Lelieveldt, B.; Vilanova, A. Cytosplore: Interactive Immune Cell Phenotyping for Large Single-Cell Datasets. *Comput. Graph. Forum* **2016**, *35*, 171–180, doi:10.1111/cgf.12893.
16. Gu, Z.; Schlesner, M.; Hübschmann, D. cola: an R/Bioconductor package for consensus partitioning through a general framework. *Nucleic Acids Res.* **2021**, *49*, e15, doi:10.1093/nar/gkaa1146.
17. Pezzotti, N.; Lelieveldt, B.P.F.; v. d. Maaten, L.; Höllt, T.; Eisemann, E.; Vilanova, A. Approximated and User Steerable tSNE for Progressive Visual Analytics. *IEEE Trans. Vis. Comput. Graph.* **2017**, *23*, 1739–1752, doi:10.1109/TVCG.2016.2570755.
18. Comaniciu, D.; Meer, P. Mean shift: a robust approach toward feature space analysis. *IEEE Trans. Pattern Anal. Mach. Intell.* **2002**, *24*, 603–619, doi:10.1109/34.1000236.
