# Supplementary material for: Landscape of Bone Marrow Metastasis in Human Neuroblastoma Unraveled by Transcriptomics and Deep Multiplex Imaging
Source: Cancers (Basel). 2021 Aug 26;13(17):4311. doi: 10.3390/cancers13174311 (PMC8431445; doi:10.3390/cancers13174311)
Supplement: Supplementary file 1 [file cancers-13-04311-s001.zip › cancers-1331004 - SUPPLMENTARY/Table_S6.pdf]

Localization of encoded proteins on cytoplasmic membrane according to Cell Atlas or Uniprot

- $\log_2$ fc DTC vs. MNC ≥ 4
- $\log_2$ fc DTC_dx vs. DTC_rel ≤ 0

1. ANK2

https://www.ncbi.nlm.nih.gov/pmc/articles/PMC5807036/

In this study, we used a SILAC approach to compare the proteomic signatures of *MYCN*-amplified IMR-32 and non-*MYCN*-amplified SK-N-SH human neuroblastoma cells.
Molecules predicted to be inhibited in the IMR-32 compared to the SK-N-SH cells included Aly/REF export factor (*ALYREF*), Ankyrin (ANK)2, autophagy related 7 (ATG7), coiled-coil domain containing 88A (*CCDC88A*), chromatin target of PRMT1 (*CHTOP*), cleavage and polyadenylation specific factor 1 (*CPSF1*), *CPSF2* and *CPSF3* among others, primarily involved in RNA post-transcriptional modification, molecular transport and RNA trafficking.

https://www.ncbi.nlm.nih.gov/pmc/articles/PMC4260775/

GLIAL ANKYRINS FACILITATE PARANODAL AXOGLIAL JUNCTION ASSEMBLY

The interaction between NFasc and ankyrins is required for aggregation of neuroblastoma cells mediated by homophilic NFasc interactions[25]

2. CNTN1

https://www.ncbi.nlm.nih.gov/pmc/articles/PMC3493082/

CNTN1 is a novel member of the contactin subgroup of the immunoglobulin superfamily which also includes contactin-2, 5 and 6. The well-known role of these proteins and ligands is the repulsive guidance of nerve axons, regulating neurite extension in a mouse neuroblastoma cell line and primary hippocampal neurons (12–14).

3. TUB

Not found in context with NB or cancer

4. ENAH

https://www.sciencedirect.com/science/article/pii/S0304383515003250

To answer the question of whether the development of cisplatin resistance involves similar type of proteins, we used Ingenuity Pathway Analysis software (IPA) to analyse the differentially expressed proteins for each individual cell line pair. The analysis for ‘molecular and cellular functions’ indicated that the top scoring function was cellular growth and proliferation, followed by cell death and survival (Fig. 2A and Table 3), both consistent with acquisition of a chemotherapeutic drug resistant phenotype. IPA analysis for canonical

pathway analysis identified the top 5 pathways for each cisplatin resistant cell line pair (Fig. 2B).

Table 3. List of proteins identified in top scoring molecular and cellular functions predicted by IPA.

Function	Name	p-value	Molecules
Cellular growth and proliferation	CHP-212Cis100	5.54E−10	HNRNPL, SRSF2, DBN1, NPM1, PFN1, S100A11, GNB2L1, SFPQ, HSPA5, TUBB, LMNB1, CACYBP, HNRNPA1, TARDBP, HNRNPF, YWHAG, CFL1, ATP5A1, RPL23A, SRSF3, CBX1, ATP5B, LMNB2, ZYX, ACTN4, VDAC1, XRCC5, HNRNPAB, RPSA, SPTBN1, PRDX1, PDIA3, HNRNPA2B1, PKM, HNRNPK, UCHL1, PHB, HSP90AB1, AHNAK, FLNA, ANXA1, VCP, VCL, NCL, HNRNPC, ACTN1, HNRNPU, ACTB, G6PD, VIM, HNRNPD, HSPD1, ACLY, HNRNPM, ENO1, KRT8, DAZAP1, SERPINH1, EIF4A1, ACAT1, MYH9
	KellyCis83	2.97E−04	CHGA, PFN1, EML4, CCT2, RAN, UBE2V2, SFPQ, TUBB, HSPA5, EIF3C/EIF3CL, STMN1, ENAH, PGK1, CDC37, EWSR1, ATP5A1, CLTC, PFDN5, HSPA8, PTBP1, ATP5B, FSCN1, XRCC5, GSTP1, RPSA, PRDX2, PEBP1, NME1, PRDX1, PDIA3, KPNA2, PKM, EEF1B2, DDX17, IGF2BP1, HNRNPK, HSP90AB1, PHB, VCP, PFN2, HNRNPU, TRIM28, VIM, HSPD1, HNRNPM, ENO1, GPI, ACAT1, CCT7, GAP43
	SK-N-ASCis24	5.04E−08	NPM1, TPD52L2, PFN1, SEPT9, NME2, UBE2V2, HSPA5, TUBB, CTSD, HNRNPA1, FASN, S100A10, PGK1, YWHAG, PLEC, LMNA, NASP, LIMA1, SPTAN1, ACTN4, CTTN, LDHA, SPTBN1, PRDX1, PDIA3, KPNA2, HNRNPA2B1, NAA10, HNRNPK, PHB, AHNAK, HNRNPR, VCL, ACTN1, ITGB1, CALR, PTMA, ACTB, ITGA2, VIM, HNRNPD, NAP1L1, HNRNPM, COL1A1, ALB, KRT8, DAZAP1, NT5E, EIF4A1, CRABP2, CD44,MYH9
Cell death and survival	CHP-212Cis100	9.59E−10	SRSF2, NPM1, S100A11, MAP1B, GNB2L1, SFPQ, HSPA5, TUBB, LMNB1, CACYBP, HNRNPA1, TARDBP, P4HB, YWHAG, CFL1, ATP5A1, HSPA9, YWHAZ, STOML2, HSP90AA1, ZYX, KRT18, ACTN4, AARS, FH, VDAC1, XRCC5, RPSA, HSPB1, HSD17B10, SPTBN1, FLNB, PDIA3, PRDX1, PKM, GAPDH, HNRNPK, PRDX6, ATP5H, UCHL1, PHB, HSP90AB1, FLNA, ANXA1, ANXA5, HSPE1,

5. CHRNA3

https://www.ncbi.nlm.nih.gov/pubmed/23417100

In the present study, we validated the ability of 14 commonly used real-time RT-PCR markers to detect MRD based on their expression in neuroblastoma TICs, and we developed a novel MRD detection protocol, which scored the samples as MRD-positive when the expression of one of the 11 real-time RT-PCR markers (CHRNA3, CRMP1, DBH, DCX, DDC, GABRB3, GAP43, ISL1, KIF1A, PHOX2B and TH) exceeded the normal range.

6. PRAME

https://www.ncbi.nlm.nih.gov/pubmed/15240516

The tumor-associated antigen PRAME is universally expressed in high-stage neuroblastoma and associated with poor outcome.

7. SRRM3

https://www.ncbi.nlm.nih.gov/pmc/articles/PMC4459148/

An alternatively spliced form of REST, called REST4, has been identified in several cancer types that do not express normal REST transcripts (e.g., neuroblastoma[9], small cell lung cancer[5,10], and breast cancer[4]). In neurons, alternative splicing of REST into REST4 is controlled by a neuronal-specific Ser/Arg repeat protein (nSR100/SRRM4). Interestingly, REST can directly silence SRRM4 expression, thereby preventing alternative splicing and thus acting as an on/off switch for neuronal gene expression[11,12]. A similar REST-dependent alternative splicing mechanism could conceivably play a role in acquisition of neuronal-like properties of breast cancer cells, enabling them to become invasive[13].

8. JPH4

https://www.ncbi.nlm.nih.gov/pubmed/19065659

JPH4 seems to be a real miR-205 target in vitro and in vivo, and a candidate tumor suppressor gene in EEC. Based on this study in EEC, miRNAs predicted to be involved in tumorigenesis and tumor progression have been identified and placed in the context of the transcriptome of EEC. This work provides a framework on which further research into novel diagnosis and treatment of EEC can be focused.

https://www.ncbi.nlm.nih.gov/pmc/articles/PMC5485426/

We also checked the expression of a related member *JPH4* in tumor cell lines and primary cases (Suppl. Figure S1). *JPH4* was frequently downregulated in tumor cells and primary tumors, while no correlation of *JPH4* and *JPH3* expression was detected. The functions of *JPH4* in cancer pathogenesis need further investigations.

9. GNAO1
10. GPRIN1

https://www.omim.org/entry/611239

in brain tissue and the central nervous system with highest expression in the spinal cord. Northern blot analysis of mouse tissues detected expression in brain only, and Western analysis detected protein in mouse neuroblastoma and rat pheochromocytoma cells. Using immunofluorescence studies and Western analysis of cell fractions, the authors found that both GPRIN1 and GNAO1 (139311) are membrane-bound proteins that are enriched in the growth cones of neurites.

11. ADCY1

https://www.ncbi.nlm.nih.gov/pmc/articles/PMC4870777/

Our model included genes that may be interesting for further research based on either their chromosomal location, their known function, or their possible role as drug targets (*ADCY1*, *AKR1C1* and *SCNA3*). Not surprisingly, this set of 18 predictive genes contains numerous genes that have been reported to have a role in the neuronal differentiation which if arrested contributes to early event in NB pathogenesis as also demonstrated by our recent work on genetic susceptibility to NB [26]. For instance, *ADCY1GNAI1* and *PRKACB* genes are associated with the cAMP-mediated signaling which plays a crucial role in initiating differentiation in transformed and embryonic cells of neuronal and glial origin [27]. cAMP-stimulating agents also induce differentiation in human and mouse NB cells [27]. *ARHGEF10L* gene is a member of the Rho family of guanine nucleotide exchange factors (GEF) that activate Rho GTPases. Interestingly, frequent mutations of RAC-RHO pathway genes regulating neuritogenesis have been found in NBs stage 3 and 4 [28]. Further genes reported to have a role in neuronal differentiation are *HOXC6* [29], *SOX4* [30], *FOXP1* [31], *GFRA3* [32], and *PTPRH* [33]. Our recent study shows the biological role of *FOXP1* in contributing to NB progression and unfavorable patient outcome [34]. This is in line with the evidence that high risk neuroblastomas are characterized by low expression of genes involved

in neuronal differentiation [15, 35–37]. Importantly, the gene network and GO analysis showed that "PKA activity", which includes *ADCY1* and *PRKACB* genes, was the most enriched biological term. *ADCY1* encodes a form of adenylate cyclase whereas *PRKACB* encodes a catalytic subunit of PKA [27]. Both genes show lower expression values in patients classified to be at high risk. Recently, adenylyl cyclases have emerged as potential drug target in diverse diseases [38] whereas PKA signaling pathway is known to antagonize Hedgehog signaling [39]. Interestingly, the activation of PKA pathway by forskolin (*ADCY1* activator) has been associated with a reduction of cell proliferation and an induction of apoptosis by inhibition of Hedgehog signal in NB cell lines [40]. Moreover, a recent study demonstrated that the neuropeptide pituitary adenylyl cyclase activating polypeptide (PACAP), another *ADCY1* activator, inhibits proliferation of primary medulloblastoma derived tumorsphere cultures by PKA activation and inhibition of Hedgehog signal [41]. Together, these data support the idea that regulation of PKA signaling by *ADCY1* activation might be an additional therapeutic strategy for stage 4 NB.

12. RGS4

https://www.ncbi.nlm.nih.gov/pubmed/10936173

Regulator of G protein signaling (RGS) proteins are GTPase-activating proteins for heterotrimeric G proteins. One of the best-studied RGS proteins, RGS4, accelerates the rate of GTP hydrolysis by all G(i) and G(q) alpha subunits yet has been shown to exhibit receptor selectivity. Although RGS4 is expressed primarily in brain, its effect on modulating the activity of serotonergic receptors has not yet been reported. In the present study, transfected BE(2)-C human neuroblastoma cells expressing human 5-HT(1B) receptors were used to demonstrate that RGS4 can inhibit the coupling of 5-HT(1B) receptors to cellular signals. Serotonin and sumatriptan were found to stimulate activation of extracellular signal-regulated kinase. This activation was attenuated, but not completely inhibited, by RGS4. Similar inhibition by RGS4 of the protein kinase Akt was also observed. As RGS4 is expressed at high levels in brain, these results suggest that it may play a role in regulating serotonergic pathways.

https://www.ncbi.nlm.nih.gov/pubmed/21712773

METHODS:
To elucidate thise role of RGS4 in pathophysiology of schizophrenia, we silenced RGS4 using siRNAs in human neuroblastoma cell lines and we studied the effects of differential RGS4 expression by microarray.

13. CADM1

https://www.ncbi.nlm.nih.gov/pubmed/18084322

Expression of the tumour suppressor gene CADM1 is associated with favourable outcome and inhibits cell survival in neuroblastoma.

Cell adhesion molecule 1 (CADM1) is a putative tumour suppressor gene, which is downregulated in many solid tumours. In neuroblastoma, loss of CADM1 expression has recently been found in

disseminated tumours with adverse outcome, prompting us to investigate its role in neuroblastoma tumour progression.

14. CDH2

https://www.ncbi.nlm.nih.gov/pmc/articles/PMC3280274/

One of the first and most important steps in the metastatic cascade is the loss of cell-cell and cell-matrix interactions. N-cadherin, a crucial mediator of homotypic and heterotypic cell-cell interactions, might play a central role in the metastasis of neuroblastoma (NB), a solid tumor of neuroectodermal origin.

15. RIMBP2

Not found in context with NB or cancer

16. PLEKHA6

https://www.ncbi.nlm.nih.gov/pmc/articles/PMC4040330/

We also observed involvement of several genes that were present in a recently developed retinoic acid-induced neuroblastoma differentiation signature: namely, *CRABP2, EGR1, ST6GAL1, PLEKHA6, MMP11* (upregulated) and *SLC25A1, SLC36A1, SLC36A4* (downregulated) (33) (Figure 5b).

17. SLC22A17

Not found in context with NB or cancer

18. BAI2

http://cancerres.aacrjournals.org/content/66/12/6050

The final cluster (G3; Supplementary Table S3) of 741 genes was enriched for those highly expressed in the more benign subsets of neuroblastoma. GO terms overrepresented included cellular communication and processing, vesicle-mediated transport, signal transduction, neurogenesis, and neurophysiologic processes. Genes known to be involved in normal sympathetic nervous system development, such as *TH, DBH, PHOX2B, GATA3*, and *NTRK1*, all showed higher expression in the nonmetastatic cases within this cluster. This cluster was also enriched for genes mapping to chromosome arm 1p, such as *STX12, PTP4A2, GNB1, CLSTN1, PUM1, RERE, CHD5*, and *BAI2*, and these showed lower expression in the metastatic cases with 1p36 LOH. The nonreceptor tyrosine kinase *FYN* also showed differential expression in a similar pattern.

19. DPP6

https://books.google.at/books?id=mQ1GAAAAQBAJ&pg=PA64&lpg=PA64&dq=DPP6+cancer&source=bl&ots=2Us-P6V8SQ&sig=9TLtc01qUgkiT4nh4A6U-QIAAx0&hl=de&sa=X&ved=0ahUKEwi39Iv9xo7aAhUrh6YKHTrmC8IQ6AEIWzAI#v=onepage&q=DPP6%20cancer&f=false

***DPP6* and *PRLHR* Genes**

The functional significance of *DPP6* (dipeptidyl-peptidase 6) and *PRLHR* (prolactin-releasing hormone receptor and their potential roles in the development of pancreatic cancer are intriguing. *DPP6* encodes a single-pass type II membrane protein that is a member of the S9B family in clan SC of the serine proteases. This protein has no detectable protease activity but binds specific voltage-gated potassium channels and alters their expression and biophysical properties. Genetic variation in *DPP6* has been associated with susceptibility to amyotrophic lateral sclerosis (van Es et al. 2008). PRLHR is a seven-transmembrane domain receptor for prolactin-releasing hormone and is a G protein-coupled receptor. Physical activity and a genetic variant of PRLHR have been associated with hypertension (Franks et al. 2004; Bhattacharyya et al. 2003).

20. PCLO

http://www.nature.com/articles/onc201715?WT.feed_name=subjects_gastrointestinal-cancer

The presynaptic cytomatrix protein Piccolo, encoded by *PCLO*, is frequently mutated and amplified in esophageal squamous cell carcinoma (ESCC), but its exact roles in ESCC remain unclear. Here we report that Piccolo expression correlates significantly with clinical stage, patient survival and tumor embolus. Functional studies demonstrate that *PCLO* knockdown remarkably attenuates ESCC malignancy *in vitro* and *in vivo*, and ectopic EGFR expression partially compensates for Piccolo loss.

21. UNC13A

Not found in context with NB or cancer

22. SYTL4

Not found in context with NB or cancer

23. DCLK1

http://journals.plos.org/plosone/article?id=10.1371/journal.pone.0075752

Silencing of Doublecortin-Like (DCL) Results in Decreased Mitochondrial Activity and Delayed Neuroblastoma Tumor Growth

We have previously proposed the Doublecortin-like kinase (*DCLK1*) gene as an attractive molecular target for NB therapy [7,9]. DCLK-derived proteins belong to doublecortin (DCX) family and include the microtubule associated proteins (MAPs) DCLK-long and doublecortin-like (DCL). DCL and DCLK-long are highly expressed in neuroblasts and are vital for neuroblast proliferation, migration and differentiation [10,11]. Silencing of DCLK-derived MAPs results in cell-cycle arrest and apoptosis in NB cells [9,11].

24. PRRT2

https://www.ncbi.nlm.nih.gov/pmc/articles/PMC5351611/

We identified *PRRT2* and *DAB2IP* to be frequently mutated in MMR deficient cell lines, colorectal and endometrial cancer patient samples. Further characterization of *PRRT2* revealed an important role of this gene in cancer biology. Both normal prostate cell lines and a colorectal cancer cell line showed

increased proliferation, migration and invasion when expressing the mutated form of PRRT2 (ΔPRRT2).

25. RGMB

https://www.ncbi.nlm.nih.gov/pubmed/26910889

Repulsive guidance molecule B inhibits metastasis and is associated with decreased mortality in non-small cell lung cancer.

RGMB was downregulated in NSCLC ($P \leq 0.001$), possibly through promoter hypermethylation. Reduced RGMB expression was observed in advanced-stage tumors ($P = 0.017$) and in tumors with vascular invasion ($P < 0.01$), and was significantly associated with poor overall survival (39 vs. 62 months, $P < 0.001$) and with disease-associated patient mortality ($P = 0.015$).

26. RNF165

https://www.ncbi.nlm.nih.gov/pmc/articles/PMC4533625/

In addition, *KPNA4, SYT1, PLCB1, SPRED1, MBNL2, RNF165, MEF2C, MBNL1, ZFP36L1 and CELF2*, were found to be likely to play significant roles in the process of metastatic prostate carcinoma.

27. NCKAP1

https://www.ncbi.nlm.nih.gov/pubmed/27432794

The WASF3-NCKAP1-CYFIP1 Complex Is Essential for Breast Cancer Metastasis.

Here, we report that silencing NCKAP1 destabilizes the WASF3 complex, resulting in a suppression of the invasive capacity of breast, prostate, and colon cancer cells. In an in vivo model of spontaneous metastasis in immunocompromized mice, loss of NCKAP1 also suppresses metastasis.

28. FAIM2

https://www.ncbi.nlm.nih.gov/pubmed/25188511

MYCN repression of Lifeguard/FAIM2 enhances neuroblastoma aggressiveness.

We report that Lifeguard (LFG/FAIM2 (Fas apoptosis inhibitory molecule 2)/NMP35) is downregulated in the most aggressive and undifferentiated tumors. Intringuingly, although LFG has been initially characterized as an antiapoptotic protein, we have found a new association with NBL differentiation.

29. VANGL2

https://www.ncbi.nlm.nih.gov/pubmed/27036398

Here we show that high expression of the PCP core genes Prickle1 and Vangl2 is associated with low-risk neuroblastoma, suppression of neuroblastoma cell growth and decreased Wnt/β-catenin signaling. Inhibition of Rho-associated kinases (ROCKs) that are important in mediating non-canonical Wnt signaling resulted in increased expression of Prickle1 and inhibition of β-catenin activity in neuroblastoma cells. In contrast, overexpression of Vangl2 in MYC immortalized neural stem cells induced accumulation of active β-catenin and decreased the neural differentiation marker Tuj1. Similarly, genetically modified mice with forced overexpression of Vangl2 in nestin-positive cells showed decreased Tuj1 differentiation marker during embryonal development.

30. SDK1

https://www.ncbi.nlm.nih.gov/pmc/articles/PMC5393505/

In total, 966 nonsynonymous somatic mutations were detected, including 40 tumors with a mean of 16 mutations per sample and one tumor with 314 mutations. Somatic mutations in ACC-associated genes included *TP53* (8/41 tumors, 19.5%) and *CTNNB1* (4/41, 9.8%). Genes with potential disease-causing mutations included *GNAS*, *NF2*, and *RB1*, and recurrently mutated genes with unknown roles in tumorigenesis comprised *CDC27*, *SCN7A*, and *SDK1*.

31. GABRB3

https://www.ncbi.nlm.nih.gov/pmc/articles/PMC4950657/

Early detection of tumor relapse/regrowth by consecutive minimal residual disease monitoring in high-risk neuroblastoma patients

The present study reports two high-risk neuroblastoma patients, whose MRD was consecutively monitored using 11 RT-qPCR markers (CHRNA3, CRMP1, DBH, DCX, DDC, GABRB3, GAP43, ISL1, KIF1A, PHOX2B and TH) during their course of treatment.

32. SORBS2

https://www.ncbi.nlm.nih.gov/pubmed/21602178

An integrative functional genomic and gene expression approach revealed SORBS2 as a putative tumour suppressor gene involved in cervical carcinogenesis

Reconstitution of SORBS2 expression resulted in a significant reduction in cell proliferation, colony formation and anchorage-independent growth in CaSki, HPKII and HaCaT cells, whereby anchorage-independent growth could only be investigated for CaSki cells. SORBS2 had no effect on cell migration.

33. SYT4

https://www.nature.com/articles/srep03544

Genomic Analyses across Six Cancer Types Identify Basal-like Breast Cancer as a Unique Molecular Entity

To better understand the biological significance of PC1 and PC2, we evaluated the top-300 genes having the largest positive and negative weights for both PCs (Fig. 1C and **Supplemental Data**). Gene weights are indicative of the relative contribution of each gene to the principal components. For PC1, the top-300 genes having the largest positive weight were found enriched for neuron differentiation (e.g. neuronal cell adhesion molecule [NRCAM] and N-cadherin [CDH2]), gliogenesis (e.g. SRY [sex determining region Y]-box 11 [SOX11]), cell-cell signaling (e.g. synaptotagmin IV [SYT4]) and synaptogenesis (e.g. neurexin 1 [NRXN1]).

34. SNAP91

http://clinchem.aaccjnls.org/content/55/7/1316

Detecting Minimal Residual Disease in Neuroblastoma: The Superiority of a Panel of Real-Time Quantitative PCR Markers

The other 5 candidate markers (*B4GALNT1*, *CHGB*, *STMN2*, *SNAP91*, and *STMN4*) showed relatively high expression levels in almost all control hematologic samples.

35. ADAM22

https://www.ncbi.nlm.nih.gov/pmc/articles/PMC3681815/

LGI1 acts as a specific extracellular ligand for the neuro-receptor ADAM22. It functions as a tumour suppressor of glioblastoma and neuroblastoma and has recently has been shown to impair proliferation and survival in HeLa cells (30-32). Both LGI1 and ADAM22 are genetically linked to epilepsy and the ligand/receptor complex has been suggested as a therapeutic target for synaptic disorders (24). Treatment of breast cancer cells resistant to either 4-OHT or letrozole, with recombinant LGI1, reduced cell migration. The anti-migratory action of LGI1 observed here is consistent with the suppression of cell invasion observed in glioma cells (30). LGI1 may function by inhibiting the extracellular disintegrin domain of ADAM22. Furthermore, in a significant cohort of breast cancer patients, ADAM22, along with SRC-1, was found to be an independent predictor of poor disease free survival. Taken together these studies provide strong evidence of ADAM22 as a mediator of metastasis and as a potential drug target for the treatment of endocrine related metastatic disease.

36. MYO1B

https://www.ncbi.nlm.nih.gov/pmc/articles/PMC4165591/

MYO1B is a motor protein controlling cell shape and participating cell migration and invasion by alter its motile properties and interaction with actin.[26] In this study, we demonstrated that ADAM15 and MYO1B are targets of miR-363 in neuroblastoma, suggesting that miR-363 represses tumor metastasis by inhibiting the expression of ADAM15 and MYO1B in neuroblastoma cells.

37. SLC6A2

http://clincancerres.aacrjournals.org/content/20/8/2182

Immunohistochemistry staining for NET expression. The neuroblastoma xenografts were collected from the imaging studies and fixed by formalin. Paraffin-embedded tissue sections (5 μm) were immunostained using the Discovery XT biomarker platform (Ventana). The primary antibody, anti-SLC6A2/NET polyclonal antibody (MBL; BMP029), was diluted at 1:100. Biotin-labeled anti-rabbit antibody (1:300; BA-1000; Vector Laboratories;) was used as the secondary antibody.

http://jnm.snmjournals.org/content/58/Supplement_2/39S.full

The norepinephrine transporter (NET) is a transmembrane protein responsible for transporting norepinephrine into the synaptic terminals of the central and peripheral nervous systems as well as neuroendocrine adrenal chromaffin cells. It is encoded by the SLC6A2 gene and is crucial for the reuptake of noradrenaline; thus, it regulates noradrenergic signaling, which

influences many physiologic processes, including behavior, mood, cognition, and the regulation of blood pressure and heart rate (1).

One of the most widely used theranostic agents targeting NET is metaiodobenzylguanidine (MIBG), a guanethidine analog of norepinephrine. 123I/131I-MIBG theranostics have been applied in the clinical evaluation and management of neuroendocrine tumors, especially in neuroblastoma, paraganglioma, and pheochromocytoma. 123I-MIBG imaging is a mainstay in the evaluation of neuroblastoma, and 131I-MIBG has been used for the treatment of relapsed high-risk neuroblastoma for several years, however, the outcome remains suboptimal.

38. SRCIN1

https://www.ncbi.nlm.nih.gov/pmc/articles/PMC5403293/

Overexpression of Srcin1 contributes to the growth and metastasis of colorectal cancer.

39. PCDH1

Not found in context with NB or cancer

40. KIF26B

https://www.ncbi.nlm.nih.gov/pubmed/23585914

High expression of KIF26B in breast cancer associates with poor prognosis.

41. SNAP25

https://www.ncbi.nlm.nih.gov/pubmed/1572061

Differentiation of rat PC-12 cells with nerve growth factor failed to alter steady-state levels of SNAP-25 protein; similar responses were seen in human SMS-KCNR neuroblastoma cells differentiated using retinoic acid. The presence of SNAP-25 in presynaptic regions of numerous neuronal subsets and in neural crest cell lines suggests that this protein subserves an important function in neuronal tissues.

42. TACC2

https://www.ncbi.nlm.nih.gov/pubmed/18980998

Exploiting gene expression profiling to identify novel minimal residual disease markers of neuroblastoma.

Based on sensitivity assays, 8 top-ranking markers were identified: CCND1, CRMP1, DDC, GABRB3, ISL1, KIF1A, PHOX2B, and TACC2. They were abundantly expressed in stage IV neuroblastoma tumors (n=20) and had low to no detection in normal marrow/blood samples (n=20). Moreover, expression of CCND1, DDC, GABRB3, ISL1, KIF1A, and PHOX2B in 116 marrows sampled after two treatment cycles was highly prognostic of progression-free and overall survival (P<0.001).

43. PARVA

https://www.ncbi.nlm.nih.gov/pmc/articles/PMC3459278/

Conversely, down-expressed correlated genes in *MYCN*+ NBs are mostly associated with apoptosis (*HRAS, CD44, MADD, ILK, SMPD1, MAPK8IP1*, and *APBB1*), cell-matrix and cell-cell adhesion (*CD44, FXC1, ILK*, and *PARVA*), and neuronal differentiation (*CD44, TH, ILK*, and *APBB1*) pathways at 11p11.2-pter.

44. KCNB1

https://www.nature.com/articles/s41598-017-00045-7.pdf?origin=ppub

Although several studies have demonstrated that regulation of KCNB1 is involved in neuronal apoptosis18, 19 and
KCNB1 mutation can result in early epileptic encephalopathy20, the role of KCNB1 in gliomas remains unknown.

https://livrepository.liverpool.ac.uk/3009569/1/200872671_Jul2017.pdf

4.2 K+ expression in primary neuroblastoma tumours and cell lines

An initial bioinformatic screen identified the 5 K_+ channel genes with the highest relative expression in primary neuroblastoma tumours: KCNQ2, KCNMA1, KCNH2, KCNG1 and KCNB1. These 5 channels were selected for *in vitro* assessment. 4 of these channels (KCNQ2, KNH2, KCNG1 and KCNB1) belong to the voltage-gated K_+ channel superfamily (Kv) and there is substantial evidence to support the oncogenic potential of Kv channels [193, 207].

45. RAB3C

https://www.nature.com/articles/1206853

The NBL subset-related genes may include those regulating neuronal growth, differentiation and apoptosis as well as those concerning generation and progression of NBL. We classified the differentially expressed genes into nine categories according to their known functions as shown in Table 4. The genes preferentially expressed in the F-subset showed an interesting profile as follows. (a) Signaling molecules: This group included certain protein kinases and protein phosphatases, among which protein kinase C zeta (PKCzeta) showed the most distinct F-subset-specific expression pattern. A small GTPase, RAB6B and a GTP-binding protein, RAB3C, functioning in the synaptic vesicles were also found in this group.

46. GRIA2

https://www.ncbi.nlm.nih.gov/pmc/articles/PMC4133641/

Gene expression analysis of several hundred glioblastoma samples revealed that a loss of GRIA2 (gene for GluR2) expression was 1 of the 38 gene changes that predict a poor prognosis in glioblastoma (Colman et al. 2010).

https://www.ncbi.nlm.nih.gov/pubmed/22644307

Identification of differentially expressed genes according to chemosensitivity in advanced ovarian serous adenocarcinomas: expression of GRIA2 predicts better survival.

47. RELN

https://www.ncbi.nlm.nih.gov/pubmed/20734148

Reduced expression of reelin (RELN) gene is associated with high recurrence rate of hepatocellular carcinoma.

The reelin (RELN) gene was detected as a pertinent tumor suppressor gene by means of this method. Of the 48 clinical samples obtained, 34 (79.2%) displayed reduced RELN expression in tumor tissue, and the expression level of tumor tissues clearly reduced compared with that of corresponding normal tissues (P = 0.002). Eighteen (37.5%) of 48 tumor tissues were found to be hypermethylated on the RELN gene promoter. Moreover, analysis of clinical data revealed an inverse correlation between RELN expression and HCC recurrence.

48. RYR2

https://www.ncbi.nlm.nih.gov/pubmed/28476886

Alteration of ryanodine receptor (RyR)-mediated calcium (Ca2+) signaling has been reported in Alzheimer disease (AD) models. However, the molecular mechanisms underlying altered RyR-mediated intracellular Ca2+ release in AD remain to be fully elucidated. We report here that RyR2 undergoes post-translational modifications (phosphorylation, oxidation, and nitrosylation) in SH-SY5Y neuroblastoma cells expressing the β-amyloid precursor protein (βAPP) harboring the familial double Swedish mutations (APPswe). RyR2 macromolecular complex remodeling, characterized by depletion of the regulatory protein calstabin2, resulted in increased cytosolic Ca2+ levels and mitochondrial oxidative stress. We also report a functional interplay between amyloid β (Aβ), β-adrenergic signaling, and altered Ca2+ signaling via leaky RyR2 channels. Thus, post-translational modifications of RyR occur downstream of Aβ through a β2-adrenergic signaling cascade that activates PKA. RyR2 remodeling in turn enhances βAPP processing. Importantly, pharmacological stabilization of the binding of calstabin2 to RyR2 channels, which prevents Ca2+ leakage, or blocking the β2-adrenergic signaling cascade reduced βAPP processing and the production of Aβ in APPswe-expressing SH-SY5Y cells. We conclude that targeting RyR-mediated Ca2+ leakage may be a therapeutic approach to treat AD.

https://www.ncbi.nlm.nih.gov/pmc/articles/PMC4941301/

Microarrays analyses provided *in vivo* evidence that cinacalcet triggers ER stress in neuroblastoma, including up-regulation of RYR2. Intracellular Ca2+ leak via RYR2 and consequent depleted ER stores have been associated with ER stress in pancreatic β cells.

49. UNC79

Not found in context with NB or cancer

50. MAGI1

https://www.ncbi.nlm.nih.gov/pubmed/24982328

Regulation and involvement in cancer and pathological conditions of MAGI1, a tight junction protein.

Furthermore, evidence has accumulated to confirm the critical role of MAGI1 in regulating cell-cell contacts, which is always disrupted in tumor progression and is associated with invasiveness and metastasis. It has also been shown in vitro that the abnormal expression of MAGI1 influences the adhesion and invasiveness of cancer cells.

51. FRRS1L

Not found in context with NB or cancer

52. SORCS1

Not found in context with NB or cancer

53. BCAR1

https://www.ncbi.nlm.nih.gov/pubmed/11585672

BRCA1-associated tumorigenesis: what have we learned from knockout mice?

Brca1 is essential in maintaining genome integrity through its involvement in DNA damage repair, G(2)-M cell-cycle checkpoint and centrosome duplication. The loss of Brca1 is not sufficient for malignant transformation, rather, it triggers multiple genetic alterations, including the inactivation of p53 and activation of a number of oncogenes, that ultimately result in mammary tumorigenesis.

54. CDH4

https://www.ncbi.nlm.nih.gov/pmc/articles/PMC5240236/

The human CDH4 gene, which encodes the R-cadherin protein, has an important role in cell migration and cell adhesion, sorting, tissue morphogenesis, and tumor genesis. This study analyzed the relationship of CDH4 mRNA expression with lung cancer.

https://www.ncbi.nlm.nih.gov/pubmed/21665361

CDH4 as a novel putative tumor suppressor gene epigenetically silenced by promoter hypermethylation in nasopharyngeal carcinoma.

Genes with low fc_DTC_dx_vs_DTC_rel (blue values)

55. ARHGEF28

https://www.ncbi.nlm.nih.gov/pubmed/25922072

Gastrin-stimulated Gα13 Activation of Rgnef Protein (ArhGEF28) in DLD-1 Colon Carcinoma Cells.

These results show that Rgnef functions as an effector of Gα13 signaling and that this linkage may mediate FAK activation in DLD-1 colon carcinoma cells.

56. GRIK2

https://www.ncbi.nlm.nih.gov/pubmed/19824040

Glutamate receptor, ionotropic, kainate 2 silencing by DNA hypermethylation possesses tumor suppressor function in gastric cancer.

Taken together, these results suggest that GRIK2 may play a tumor-suppressor role in gastric cancer.

57. GFRA3

https://www.ncbi.nlm.nih.gov/pmc/articles/PMC5536348/

Quantitative reverse-transcription PCR (qRT-PCR) revealed significant upregulation of neuronal differentiation genes, including *Gfra3* and *Ntn3* (Figure 2F), which are known to be upregulated in neuroblastoma cells undergoing neuronal differentiation (Mao et al., 2011; Wang et al., 2013; Wang et al., 2014).

https://www.ncbi.nlm.nih.gov/pubmed/28871274

Paired Expression Analysis of Tumor Cell Surface Antigens.

Specifically, we found that, for MYCN amplified neuroblastoma, pairwise expression of ACVR2B or anaplastic lymphoma kinase (ALK) with GFRA3, GFRA2, Cadherin 24, or with one another provided the strongest hits. For MYCN, non-amplified stage 4 neuroblastoma, neurotrophic tyrosine kinase 1, or ALK paired with GFRA2, GFRA3, SSK1, GPR173, or with one another provided the most promising paired-hits. We propose that targeting these markers together would increase the specificity and thereby the safety of CAR-based therapy for neuroblastoma.

58. SHANK2

http://cancerres.aacrjournals.org/content/75/15_Supplement/475

Identification of SHANK2 as a tumor suppressor disrupted by recurrent somatic structural variation (SV) in neuroblastoma

Low SHANK2 expression in primary tumors obtained at diagnosis was associated with worse overall survival ($p = 6.3 \times 10^{-5}$), suggesting SHANK2, known to regulate neuronal differentiation, may function as a tumor suppressor.

59. MPDZ

https://www.ncbi.nlm.nih.gov/pmc/articles/PMC4241148/

Mpdz RNAi efficacy in mouse neuroblastoma cells

Table 1 summarizes the results for five *Mpdz* shRNAs tested for their efficacies to affect endogenous *Mpdz* expression in mouse NS20Y cells compared to a scrambled control. This validated negative control engages the RNAi pathway but does not target any sequence in the mouse genome. The TRCN0000103482 plasmid demonstrated the highest *in vitro* knockdown of *Mpdz* expression (i.e., a 77% reduction relative to the scrambled control), and was therefore utilized for the subsequent *in vivo* RNAi experiments.

60. CNTN2

https://www.researchgate.net/publication/322267973_Tumorigenic_proteins_upregulated_in_the_MYCN-amplified_IMR-32_human_neuroblastoma_cells_promote_proliferation_and_migration

Tumorigenic proteins upregulated in the MYCN-amplified IMR-32 human neuroblastoma cells promote proliferation and migration

Lastly, L1-CAM focused network analysis revealed its strong interaction with neural cell adhesion molecule 1 (NCAM1), neurocan (NCAN), ezrin (EZR), RDX, ANK1, ANK2, contactin 2 (CNTN2), neuro-pilin 1 (NRP1), epidermal growth factor receptor (EGFR) and RAN binding protein 9 (RANBP9) (Fig. 3B), most of which drive angiogenic and migratory processes to fuel cellular inva-sion, migration and metastatic spread.

61. PTCHD1

https://www.omim.org/entry/300828

Fluorescence-tagged PTCHD1 was expressed at the cell membrane in transfected COS-7 and SK-N-SH human neuroblastoma cells. Noor et al. (2010) also identified noncoding antisense RNAs that overlap the PTCHD1 gene. Fluorescence-tagged PTCHD1 was expressed at the cell membrane in transfected COS-7 and SK-N-SH human neuroblastoma cells. Noor et al. (2010) also identified noncoding antisense RNAs that overlap the PTCHD1 gene. Fluorescence-tagged PTCHD1 was expressed at the cell membrane in transfected COS-7 and SK-N-SH human neuroblastoma cells. Noor et al. (2010) also identified noncoding antisense RNAs that overlap the PTCHD1 gene. Fluorescence-tagged PTCHD1 was expressed at the cell membrane in transfected COS-7 and SK-N-SH human neuroblastoma cells. Noor et al. (2010) also identified noncoding antisense RNAs that overlap the PTCHD1 gene.

62. FNDC5

https://www.ncbi.nlm.nih.gov/pubmed/28377712

PGC-1α or FNDC5 Is Involved in Modulating the Effects of Aβ1-42 Oligomers on Suppressing the Expression of BDNF, a Beneficial Factor for Inhibiting Neuronal Apoptosis, Aβ Deposition and Cognitive Decline of APP/PS1 Tg Mice.

63. ARHGAP36

http://www.nature.com/articles/ncomms12963/figures/8

ARHGAP36 is expressed in neuroblastoma cells and promotes aberrant activation of the Hedgehog pathway

http://berlin-buch.com/de/news/new.php?we_objectID=3795

Abnormal viel ARHGAP36 findet sich außerdem bei mindestens einem der vier Subtypen des Medulloblastoms, der am meisten verbreiteten Art von Hirntumoren bei Kindern. Auch beim Neuroblastom, einer weiteren häufigen Krebserkrankung des Nervensystems bei Kindern, ist dieses Protein in den Zellen in unnatürlich hoher Zahl vorhanden. Die genaue biologische Rolle von ARHGAP36 ist noch nicht verstanden, aber es liegt nahe, dass es an der Muskelentwicklung und bei manchen Krebsarten am Tumorwachstum beteiligt ist. So könnten Änderungen im PKA-Signalsystem bei vielen Krebsarten das Tumorwachstum beeinflussen.

64. BAI3

https://www.sciencedirect.com/science/article/pii/S0014579304007318

Expression of BAI3 was generally decreased in malignant gliomas, whereas angiogenic genes, such as VEGF and HIF-1α were increased. In the real-time RT-PCR analyses, the relative expression levels of BAI1 in normal brain tissue and SHSY5Y neuroblastoma cells were highest among BAIs, and the generally decreased expressions of BAIs in high-grade glioma compared to normal tissue (higher cycle-threshold values in high-grade glioma than normal brain) were observed (data not shown) [26].

65. LSAMP

http://www.thesis.bilkent.edu.tr/0006320.pdf

ANALYSIS OF LSAMP GENE AS A TUMOR SUPPRESSOR IN NEUROBLASTOMA

66. ARHGAP29

https://www.ncbi.nlm.nih.gov/pmc/articles/PMC4951313/

Serum-circulating miRNAs predict neuroblastoma progression in mouse model of high-risk metastatic disease

Conversely, for '*upregulated homologous miRNAs*' BCL2L11, BCL11B, ABL2, BCL11A, ABCA1, ACVR2B, ADAM12, AIF1L, ARHGAP29, ASPH, BET1L, C2orf69, CALM1, CALN1, CCND2, ACAP2, AGO1, ANKRD13A, ANKRD13C, ARHGAP21, ARHGEF18, ASXL2, ATAD3C, ATP5J2-PTCD1, ATP6V1A, BACH2, BDNF, BHLHE22, C7orf43, CELSR3, CNBP, and CCND1 constitute the common gene targets (Supplementary Table 2).

67. MMP16

https://www.ncbi.nlm.nih.gov/pmc/articles/PMC5641122/

MMP16 promotes tumor metastasis and indicates poor prognosis in hepatocellular carcinoma

https://www.ncbi.nlm.nih.gov/pubmed/28927056

Membranous type matrix metalloproteinase 16 induces human prostate cancer metastasis.

https://www.ncbi.nlm.nih.gov/pmc/articles/PMC5239520/

MMP16 is a marker of poor prognosis in gastric cancer promoting proliferation and invasion

68. GCGR

https://www.ncbi.nlm.nih.gov/pmc/articles/PMC5203805/

Oxyntomodulin (OXM) is a proglucagon-derived peptide that co-activates the GLP-1 receptor (GLP-1R) and the glucagon receptor (GCGR). The neuroprotective action of OXM, however, has not been thoroughly investigated. In this study, the neuroprotective effect of OXM was first examined in human neuroblastoma (SH-SY5Y) cells and rat primary cortical neurons.

69. CNTNAP4

https://www.ncbi.nlm.nih.gov/pubmed/18398821

The area most frequently deleted resided on 16q23.1, 3.5 MB downstream of the area most significantly associated with survival, and included the tumor suppressor gene ADAMTS18 and the cell recognition gene CNTNAP4.

70. ELN

https://www.ncbi.nlm.nih.gov/pmc/articles/PMC3664071/

Several constituents of the extracellular matrix or membrane proteins involved in cell adhesion, motility or proliferation that map to chromosome 7, namely *PTN*, *CNTNAP2*, *ELN*, *HSPB1*, *SEMA3E*, and *COL1A2*, were upregulated in the 11q-deleted group (n=8). In the same group of tumors, *CD44* was the top upregulated gene on 11p.

71. DSEL

https://www.ncbi.nlm.nih.gov/pmc/articles/PMC2958872/

The chondroitin/dermatan sulfate synthesizing and modifying enzymes in laryngeal cancer: Expressional and epigenetic studies

We identified that many enzymes were expressed in the cancerous specimens intensively. Dermatan sulfate epimerase was expressed exclusively in the cancerous parts and in minor amounts in healthy tissues; in the macroscopically normal samples it was not detected.

72. NTNG1

Not found in context with neuroblastoma or cancer

73. COL16A1

Not found in context with NB or cancer

74. CDH6

https://www.ncbi.nlm.nih.gov/pmc/articles/PMC4050695/

Cadherin 6 promotes neural crest cell detachment via F-actin regulation and influences active Rho distribution during epithelial-to-mesenchymal transition

Neural crest cells (NCCs) undergo EMT from the neuroepithelium during normal development and display several changes in cadherin expression, making them ideal for studying cadherin-switching functions in EMT. Because NCC derivatives can give rise to neuroblastoma and melanoma, elucidation of NCC EMT mechanisms is important for understanding the biology of these cancers.

75. LAMA4

https://www.ncbi.nlm.nih.gov/pmc/articles/PMC3702642/

The complete list of mRNAs was submitted to GSEA and the top enriched gene set was the ECM structural constituents with a nominal p-value of 0.008 (Figure 5). This gene set included upregulation in expression of Tfpi2, LAMA4 (laminin alpha 4 (30, 31)), FBLN2 (fibulin 2), (32), PRELP (ECM protein that functions to anchor basement membranes to the underlying connective tissues (33)), COL4A2 (Collagen type 4 alpha 2, (34, 35) (Figure 5). mRNA analysis was done also on a group of 10 deficient caspase-8 mice that did not show metastasis to BM by histology (Figure 4B).

76. CDH8

http://onlinelibrary.wiley.com/doi/10.1002/ijc.20092/full

Olf/EBF proteins are expressed in neuroblastoma cells: Potential regulators of the *Chromogranin A* and *SCG10* promoters

Few direct targets have been defined in other compartments of the nervous system, even though the expression of retinoic acid protein 1 and cadherin 8 is defective in cells migrating from the subventricular zone in the lateral ganglionic eminence to the mantle zone in *O/E-1* deficient mice.

https://peerj.com/articles/3175/

Profiling transcriptomes of human SH-SY5Y neuroblastoma cells exposed to maleic acid

According to our previous transcriptome and enrichment analysis, calcium binding was inferred to be affected by maleic acid. Among the differentially expressed genes, eight genes

are associated with the GO term of calcium binding (GO:0005509) including S100A3, GNPTAB, CDH23, PCDHAC1, PADI1, CRACR2B, MAN1A2, and CDH8.

77. GPR179

https://www.ncbi.nlm.nih.gov/pmc/articles/PMC4333349/

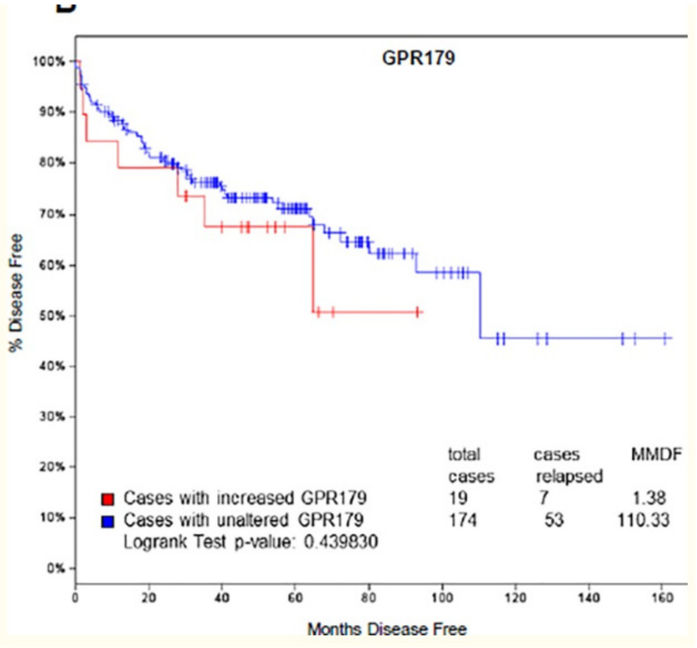

Very recently, GPR179, another orphan GPCR in the same family also called GPR158-like, was identified as a possible AR regulator in a genome-wide RNA interference screen [52]. Thus, we also asked whether increased GPR179 mRNA expression (22 out of 216 cases) was similarly associated with disease-free survival in PCa patients. Our analysis showed that increased GPR179 expression was not associated with changes in disease-free survival (p = 0.439; Fig. 10B).

78. SLCO1A2

http://www.abcam.com/slco1a2-antibody-ab221804.html

Images –

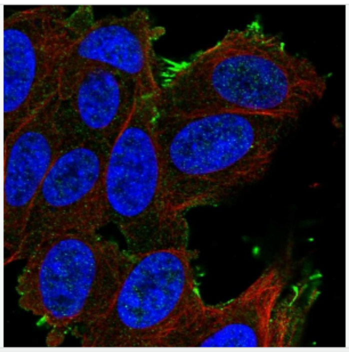

Immunocytochemistry/ Immunofluorescence - Anti-SLCO1A2 antibody (ab221804)

PFA-fixed, Triton X-100 permeabilized SH-SY5Y (human neuroblastoma cell line from bone marrow) cells stained for SLCO1A2 (green) using ab221804 at 4 µg/ml in ICC/IF.

79. KCNH1

https://www.ncbi.nlm.nih.gov/gene/3756

Overexpression of the gene may confer a growth advantage to cancer cells and favor tumor cell proliferation.

https://www.ncbi.nlm.nih.gov/pubmed/24062569

KCNH1 potassium channels are expressed in cervical cytologies from pregnant patients and are regulated by progesterone.

80. SCN7A

https://www.ncbi.nlm.nih.gov/pubmed/19326446

Identification and functional characterization of the promoter of the mouse sodium-activated sodium channel Na(x) gene (Scn7a).

Na(x) is a sodium channel, thought to be a descendant of the voltage-gated sodium channel family. Nevertheless, Na(x) is not activated by voltage but rather by augmentation of extracellular sodium over 150 mM. In the brain, it is localized to the circumventricular organs, important regions for salt and water homeostasis in mammals, where it operates as a sodium-level sensor of body fluid. Na(x) channel is expressed in lung, uterus, and heart, and it is also found in trigeminal and dorsal root ganglia and in nonmyelinating Schwann cells, where its physiological role remains unclarified. Here we identified the promoter and transcription start sites of Na(x) sodium channel in dorsal root ganglia neurons from mouse. We report a characterization of the basal TATA-less promoter and the sequence requirements for promoter activity in Neuro 2A neuroblastoma cells and in dorsal root ganglia neurons, where basal promoter activity seems to require NGFI-C and Ebox DNA elements. Finally, we provide evidence that a repression mechanism that inhibits Na(x) expression may be present in certain tissues. These findings provide the basis with which to understand tissue-specific regulation of Na(x) sodium channel gene (Scn7a) expression.

81. LRRC4C

https://molecular-cancer.biomedcentral.com/articles/10.1186/1476-4598-13-266

A genomic database analysis has since identified that LRRC4 is a member of the LRRC4 (NGL, netrin-G ligand) family and belongs to the superfamily of LRR proteins. Moreover, there are three known members in the LRRC4 family; LRRC4C (NGL-1), LRRC4 (NGL-2) and LRRC4B (NGL-3) [10]. LRRC4/NGL-2 displays down-regulation or expression deletion in primary brain tumor biopsies and has the potential to suppress brain tumor growth.

82. GFRA1

https://www.ncbi.nlm.nih.gov/pubmed/12095931

We also analyzed markers located close to several candidate genes (RET, NF1, GDNF, GFRA1, EDNRB, and EDN3) involved to a different extent in other neurocristopathies. Our findings indicate that the candidate chromosomal regions and genes analyzed are not in linkage with neuroblastoma.

83. GRIK3

https://www.ncbi.nlm.nih.gov/pubmed/28631555

GRIK3: A novel oncogenic protein related to tumor TNM stage, lymph node metastasis, and poor prognosis of GC.

Furthermore, additional experiment showed that the lymph node metastasis tissues had higher GRIK3 expression than their matched primary GC tissues.

84. UBE2QL1

https://www.ncbi.nlm.nih.gov/pmc/articles/PMC4028990/

UBE2QL1 is Disrupted by a Constitutional Translocation Associated with Renal Tumor Predisposition and is a Novel Candidate Renal Tumor Suppressor Gene

We identified an uncharacterized gene, *UBE2QL1*, that was disrupted by a t(5;19)(p15.3;q12) associated with a familial predisposition to RCC, and demonstrated that *UBE2QL1* has tumor suppressor activity and is inactivated in a subset of sporadic RCC by promoter region hypermethylation and/or deletions.

85. PTPN3

https://www.ncbi.nlm.nih.gov/pmc/articles/PMC4491873/pdf/12864_2015_Article_1642.pdf

Reorganization of metastamiRs in the evolution of metastatic aggressive neuroblastoma cells.

Immunoblotting and TMA-IHC analyses revealed alterations in the expression/phosphorylation of metastamiRs' targets, including ADAMTS-1, AKT1/2/3, ASK1, AURKβ, Birc1, Birc2, Bric5, β-CATENIN, CASP8, CD54, CDK4, CREB, CTGF, CXCR4, CYCLIN-D1, EGFR, ELK1, ESR1, CFOS, FOSB, FRA, GRB10, GSK3β, IL1α, JUND, kRAS, KRTAP1, MCP1, MEGF10, MMP2, MMP3, MMP9, MMP10, MTA2, MYB, cMYC, NF2, NOS3, P21, pP38, PTPN3, CLEAVED PARP, PKC, SDF-1β, SEMA3D, SELE, STAT3, TLR3, TNFα, TNFR1, and VEGF in aggressive cells ex vivo and in a manifold of metastatic tumors in vivo.

86. FLRT3

https://www.spandidos-publications.com/mmr/9/6/2411

Unc5 receptors are involved in vasculogenesis and apoptosis. Unc5B and Unc5D were identified to interact with high-affinity fibronectin leucine rich transmembrane protein 3 (FLRT3) (18). FLRT3 and Unc5B functionally interact in modulating cell adhesion during early Xenopus development, and the effect of Unc5B on adhesion is mediated by the Rho family GTPase 1. Additionally, it has been reported that subventricular expressed transcript 1 (Svet1) contains a high proportion of repetitive sequences and maps in the first intron of Unc5D. The previously reported 'SVZ-specific expression of the Svet1 RNA' indicates putative involvement of Unc5D signaling in the multipolar migrating cells (19). Therefore, certain effects observed in these studies may be due to Svet1, and Svet1 may be upregulated

by p53 alone with Unc5D. Additionally, Unc5s can regulate the hepatocyte growth factor/methoprene-tolerant (MET) signaling pathway via an interaction with the intracellular domain of the MET receptor. The MET receptor has a dual anti-apoptotic and pro-apoptotic role in different cell types. While no ligand is bound to MET, the activated MET induces phosphatidylinositol 3-kinase-Akt-dependent signaling leading to the anti-apoptotic response. When no ligand is bound to MET, the receptor is subjected to caspase-dependent cleavage leading to the formation of a pro-apoptotic fragment of MET (20). However, the reason the cells require redundant functions of the different Unc5 proteins, requires further investigation and the identification of other associated proteins in order to elucidate how this transmembrane receptor exerts its cellular functions.

Neuroblastoma treatment is a clinical challenge. Although there have been improvements in chemotherapy, radiotherapy and drug-induced differentiation, even with transplantation, the long-term survival rate of neuroblastoma remains low. Therefore, the identification of novel genes is a prospective way for targeting treatment. Unc5D is a newly identified dependence receptor for netrin-1, and a direct target of p53. Targeting at the Unc5D gene and p53-dependent apoptosis may provide a novel strategy for neuroblastoma treatment.

87. EPHA7

https://www.researchgate.net/figure/Bmi1-has-a-critical-role-in-neuroblastoma-tumor-initiation-a-b-Primary-ganglia-tissue_fig7_230711646

Bmi1 has a critical role in neuroblastoma tumor initiation.

https://www.researchgate.net/publication/306096511_The_putative_tumor_suppressor_gene_EphA7_is_a_novel_BMI-1_target

The putative tumor suppressor gene EphA7 is a novel BMI-1 target

88. GABRG2

https://www.ncbi.nlm.nih.gov/pubmed/27733149

RNA binding protein Nova1 promotes tumor growth in vivo and its potential mechanism as an oncogene may due to its interaction with GABAA Receptor-γ2

Nova1's potential mechanism as an oncogene may due to its interaction with $GABA_A$ Rγ2

89. KCNS3

https://www.ncbi.nlm.nih.gov/pmc/articles/PMC4480740/

Silencing of voltage-gated potassium channel K_V9.3 inhibits proliferation in human colon and lung carcinoma cells

K_V9.3 (*KCNS3*) is an electronically silent K_V α-subunit that does not form electronically functional channels when expressed as a homomultimer [30].

90. CDH7

https://www.ncbi.nlm.nih.gov/pubmed/24123354

Stage 4 neuroblastomas have a high rate of local and metastatic relapse and associated disease mortality. The central nervous system (CNS) is currently one of the most common isolated relapse sites, yet the genomic alterations that contribute to these metastases are unknown. This study sought to identify recurrent DNA copy number alterations (CNAs) and target genes relating to neuroblastoma CNS metastases by studying 19 pre-CNS primary tumors and 27 CNS metastases, including 12 matched pairs. SNP microarray analyses revealed that MYCN amplified (MYCNA) tumors had recurrent CNAs different from non-MYCNA cohorts. Several CNAs known to be prevalent among primary neuroblastomas occurred more frequently in CNS metastases, including 4p-, 7q+, 12q+, and 19q- in non-MYCNA metastases, and 9p- and 14q- irrespective of MYCNA status. In addition, novel CNS metastases-related CNAs included 18q22.1 gains in non-MYCNA pre-CNS primaries and 5p15.33 gains and 15q26.1→tel losses in non-MYCNA CNS metastases. Based on minimal common regions, gene expression, and biological properties, TERT (5p), NR2F2 (15q), ALDH1A3 (15q), CDKN2A (9p), and possibly CDH7 and CDH19 (18q) were candidate genes associated with the CNS metastatic process.

91. PRR26

http://www.oncotarget.com/index.php?journal=oncotarget&page=article&op=view&path[]=19627&path[]=62707

A multivariate Cox proportional hazards regression analysis indicated that FMO6P and PRR26 showed a significant prognostic value for LUSC patients'' survival.

Since then, we determined that significantly differential expression of two novel lncRNAs (FMO6P and PRR26) could be a novel independent risk factor for LUSC. Moreover, the risk score based on these two lncRNAs could be a new indicator for the prognosis of LUSC patients.

Furthermore, the PRR26-related genes were enriched in Transcriptional misregulation in cancer, the MAPK signaling pathway, in tight-junction Proteoglycans in cancer, and in Protein digestion and absorption, most of which are classical signaling pathways closely related to the genesis and progression of cancer. For example, the MAPK signaling pathway has been proposed to be associated with the occurrence, invasion, and metastasis of LUSC [50]. Therefore, functional enrichment analysis may elucidate the role of FMO6P and PRR26 in carcinogenesis of LUSC.

92. PCDH20

https://www.ncbi.nlm.nih.gov/pubmed/16651412

Frequent silencing of the candidate tumor suppressor PCDH20 by epigenetic mechanism in non-small-cell lung cancers.

Moreover, restoration of PCDH20 expression in NSCLC cells reduced cell numbers in colony formation and anchorage-independent assays. These results suggest that epigenetic silencing by hypermethylation of the CpG-rich promoter region of PCDH20 leads to loss of PCDH20 function, which may be a factor in the carcinogenesis of NSCLC.

93. LHFPL5

Not found in context with neuroblastoma or cancer

94. NPY2R

https://www.ncbi.nlm.nih.gov/pmc/articles/PMC2955165/

Neuroblastomas are pediatric tumors which develop from sympathetic precursors and express neuronal proteins, such as neuropeptide Y (NPY). NPY is a sympathetic neurotransmitter acting via multiple receptors (Y1-Y5R). Both NPY and Y2Rs are commonly expressed in neuroblastoma cell lines and tissues. The peptide secreted from neuroblastomas stimulates tumor cell proliferation and angiogenesis. Since both processes are Y2R-mediated, the goal of this study was to assess Y2R as a potential therapeutic target for neuroblastoma. *In vitro*, Y2R antagonist (BIIE0246) prevented activation of p44/42 MAPK induced by endogenous NPY, which resulted in decreased proliferation and induction of Bim-mediated apoptosis. Similar growth-inhibitory effects were achieved with NPY siRNA and Y2R siRNA. *In vivo*, Y2R antagonist significantly inhibited growth of SK-N-BE(2) and SK-N-AS xenografts, which was associated with decreased activation of p44/42 MAPK, as well as reduced proliferation (Ki67) and increased apoptosis (TUNEL). The Y2R antagonist also exerted an anti-angiogenic effect. *In vitro*, it reduced the proliferation of endothelial cells induced by neuroblastoma-conditioned media. Consequently, the Y2R antagonist-treated xenografts had decreased vascularization and a high degree of focal fibrosis. In human neuroblastoma tissues, the expression of Y2R was observed in both tumor and endothelial cells, while NPY was predominantly expressed in neuroblastoma cells. In summary, Y2R is a promising new target for neuroblastoma therapy affecting both cancer cells and tumor vasculature.

95. PTGER1

https://www.ncbi.nlm.nih.gov/pubmed/20858737/

Prostaglandin E receptor EP1 suppresses breast cancer metastasis and is linked to survival differences and cancer disparities.

These studies support the hypothesis that EP1 functions as a metastasis suppressor and that loss of nuclear EP1 is associated with poorer overall survival and may contribute to disparities in outcome in different populations.

96. SLC5A12

Not found in context with neuroblastoma or cancer

97. CDH18

https://www.ncbi.nlm.nih.gov/pmc/articles/PMC4399759/

The genes that were highly expressed in the upper part of the colonic crypt and induced by interruption of Wnt/β-catenin signaling were *p21*, *BMP2*, *MAD*, and *CDH18* (61). The active Wnt/β-catenin pathway at the lower compartment of the colonic crypt contributes to the enhanced proliferation of the stem cell–like cells responsible for generating the epithelial progeny of the upper parts

https://www.ncbi.nlm.nih.gov/pubmed/21128281

Identification of candidate predisposing copy number variants in familial and early-onset colorectal cancer patients.

We employed genome-wide copy number profiling using high-resolution SNP arrays on germline DNA, which resulted in the identification of novel copy number variants (CNVs) in six patients (15%) encompassing, among others, the cadherin gene CDH18, the bone morphogenetic protein antagonist family gene GREM1, and the breakpoint cluster region gene BCR.

98. PCDHB8

https://www.ncbi.nlm.nih.gov/pmc/articles/PMC4359221/

In addition to the hotspot *RQCD1* mutation, we also identified recurrent mutations in the *PCDHB8* (E311K, *n* = 3), *VWA3B* (V358G, *n* = 3) and *ZNF208* (H1219Y, *n* = 3) genes. Subsequent investigation of other melanoma genomic datasets could not confirm the recurrence of these mutations.

99. KCNK2

https://www.ncbi.nlm.nih.gov/pubmed/27397543/

Over-expressed human TREK-1 inhibits CHO cell proliferation via inhibiting PKA and p38 MAPK pathways and subsequently inducing G1 arrest.

https://www.ncbi.nlm.nih.gov/pubmed/25962960

Prognostic significance of the TREK-1 K2P potassium channels in prostate cancer.

Our results suggest that TREK-1 might be a biomarker in CRFS judgment of PCa, as well as a potential therapeutic target.
